# RTK/ERK Pathway under Natural Selection Associated with Prostate Cancer

**DOI:** 10.1371/journal.pone.0078254

**Published:** 2013-11-04

**Authors:** Yang Chen, Xianxiang Xin, Jie Li, Jianfeng Xu, Xiaoxiang Yu, Tianyu Li, Zengnan Mo, Yanling Hu

**Affiliations:** 1 Center for Genomic and Personalized Medicine, Guangxi Medical University, Nanning, Guangxi Zhuang Autonomous Region, China; 2 Medical Research Center, Guangxi Medical University, Nanning, Guangxi, China; 3 Department of Urology and Nephrology, The First Affiliated Hospital of Guangxi Medical University, Nanning, China; 4 Department of Urology, the 303rd Hospital of Chinese People's Liberation Army, Nanning, Guangxi Zhuang Autonomous Region, China; 5 Research Center for Guangxi Reproductive Medicine, First Affiliated Hospital of Guangxi Medical University, Guangxi Zhuang Autonomous Region, China; 6 Fudan Institute of Urology, Huashan Hospital, Fudan University, Shanghai, China; 7 Fudan Center for Genetic Epidemiology, School of Life Sciences, Fudan University, Shanghai, China; 8 State Key Laboratory of Genetic Engineering, School of Life Sciences, Fudan University, Shanghai, China; 9 Center for Cancer Genomics, Wake Forest University School of Medicine, Winston-Salem, North Carolina, United States of America; II Università di Napoli, Italy

## Abstract

Prostate cancer (PCa) is a global disease causing large numbers of deaths every year. Recent studies have indicated the RTK/ERK pathway might be a key pathway in the development of PCa. However, the exact association and evolution-based mechanism remain unclear. This study was conducted by combining genotypic and phenotypic data from the Chinese Consortium for Prostate Cancer Genetics (ChinaPCa) with related databases such as the HapMap Project and Genevar. In this analysis, expression of quantitative trait loci (eQTLs) analysis, natural selection and gene-based pathway analysis were involved. The pathway analysis confirmed the positive relationship between PCa risk and several key genes. In addition, combined with the natural selection, it seems that 4 genes (EGFR, ERBB2, PTK2, and RAF1) with five SNPs (rs11238349, rs17172438, rs984654, rs11773818, and rs17172432) especially rs17172432, might be pivotal factors in the development of PCa. The results indicate that the RTK/ERK pathway under natural selection is a key link in PCa risk. The joint effect of the genes and loci with positive selection might be one reason for the development of PCa. Dealing with all the factors simultaneously might give insight into prevention and aid in predicting the success of potential therapies for PCa.

## Introduction

As one of the most destructive cancers worldwide [1], prostate cancer (PCa) is increasing according to recent cancer statistics in the United States [2]–[4]. Although the death rates continuing to decline (declining by 1.9% from 2000 to 2008 per year), 238,590 new cases might be discovered, with a mortality rate of 12.5% [5]. In Europe, the incidence has tripled in the last 40 years, with about 328,000 men diagnosed with prostate cancer in 2008 [6]. In order to identify the potential role of genetic factors in the development of PCa, several recent studies have been based on analysis at the gene level. Recently, one of the most effective methods, a genome-wide association study (GWAS), was used to conduct high-throughput SNP polymorphism analysis that could reveal significant loci that influence the disease. However, at the same time, with its strict standards and SNP-based analysis, some potential useful information might be ignored. So conducting the further study, which could reveal more comprehensive information, seemed to be important.

Considering the complexity of the factors inducing disease, especially cancer, the interlacing signaling pathways and cell signal transmission involved in biological activities such as cell survival, development, and apoptosis [7]–[9] might make the pathogenesis clearer. As one of the crucial cancer pathways, the RTK/ERK pathway is said to be one of the important links in cancer's development, containing a number of genes (EGFR, EGF, CCND1, MAPK3, etc.). In this pathway, receptor tyrosine kinases (RTKs) represent enzyme-linked receptors, which regulate cell proliferation and differentiation, promote cell migration and survival and modulate cellular metabolism in response to extracellular cues [10]–[12]. However, in order to arouse most RTK pathway functions, the essential effector mitogen-activated protein kinase (MAPK) cascade needs to be activated, composed of the Raf, MEK, and extracellular signal-regulated kinase (ERK) kinases (known as the ERK cascade). With activation of the MAPK cascade, a signal from a receptor on the surface of the cell to the DNA in the nucleus of the cell is transferred by proteins influencing regulation, transcription, and synthesis of the genes [13], [14]. So the growth of an organism, angiogenesis, and carcinogenesis are all involved with the critical functions of the RTK pathway.

PCa is one of the most common malignancies in the world. Its high rate of morbidity and mortality bring about enormous losses. So studying the pathogenesis of PCa seems extremely urgent, including determining which genetic factors might be significant in the process. Summarizing recent studies, the pathway might be a vital factor in the development and progression of PCa. In 2007, Caraglia M et al. [15] found that zoledronic acid (ZOL) and R115777 farnesyltransferase inhibitor (FTI, Zarnestra) were synergistic in destroying the Ras/Erk and Akt survival pathways and decreasing the phosphorylation of both mitochondrial bcl-2 and bad proteins, and caspase activation, which led to both growth inhibition and apoptosis in prostate adenocarcinoma cells. Moreover, Milone et al. [16] studied the ZOL further, which suggested the p38-MAPK pathway played a key role in inducing a more aggressive and invasive phenotype and resisting to the ZOL. In addition, our previous path enrichment and gene meta-analysis by Gene Set Enrichment Analysis (GSEA) [17] suggested that significantly expressed genes were located on the RTK/ERK pathway.

Meanwhile, recent studies have discovered natural selection in genes and networks based on populations [18]–[19], which gave a new view at the evolutionary level into the pathogenesis of diseases. However, no related studies have revealed the potential relationship between the RTK/ERK pathway under natural selection and PCa risk. Prompted by this, the present study was conducted.

This study was based on the key role function of the RTK/ERK pathway, the HapMap database and our own GWA study data about PCa risk. In this analysis, we explored the pathway via several methods: (1) association analysis between gene polymorphisms in the RTK-ERK pathway and PCa; (2) gene-based pathway testing; and (3) natural positive selection and expression quantitative trait loci (eQTLs) analysis. Combining all of these effective analyses, this study might give us a more comprehensive understanding of the RTK/ERK pathway based on genes and SNP analysis, which would help us discover potential crucial loci or genes and give a wide view on the treatment, prevention, and forecasting of Pca.

## Materials and Methods

### Study subjects

The analyses in this study were mainly based on the Chinese Consortium for Prostate Cancer Genetics (ChinaPCa) GWAS, the HapMap Project (http://www.hapmap.org), and other databases (NCBI36). Subjects in the ChinaPCA were male Han Chinese recruited from the southeastern in China. They came from different areas, including Shanghai, Suzhou, Guangxi, Nanjing, etc. There were 1417 hospital-based cases that were confirmed to have primary prostate cancer histologically and 1008 health controls that had undergone routine physical examination in local hospitals. Their characteristics are showed in the Table 1. A blood sample was obtained from each subject for DNA extraction when consent was obtained. On the basis of our pathway analysis, 50,459 polymorphic SNPs were potentially involved. All genes were extended 100 kb from beginning to end in order to ensure all loci in the pathway were included. Comprehensive epidemiological and clinical data were collected with standardized questionnaires and from the associated PCa diagnosis. Essential information was included, such as age, the level of the PSA, the record of the cancer, and other related datum. All the details were given in Jianfeng Xu et al. [20].

**Table 1:** Characteristics of the subjects included in this analysis.

	case (n = 1417)	control (n = 1008)
Age (mean±SD)	71.3±8.1	62.1±1.0
Classification of PSA, n (%)		
≤4.0	55 (4.1%)	965 (95.9%)
>4.0, <10	186 (13.9%)	32 (3.2%)
≥10	1098 (82.0%)	9 (0.9%)
Missing	78	2
Gleason score, n (%)		
<7	355 (26.3%)	NA
≥7	993 (73.7%)	NA
Missing	69	NA
Clinical stage, n (%)		
Stage (0, I, II)	727 (59.7%)	NA
Stage (III, IV)	490 (40.3%)	NA
N-stage(0)	759 (67.3%)	NA
N-stage(≥1)	369 (32.7%)	NA
M-stage(0)	810 (64.8%)	NA
M-stage(≥1)	440 (35.2%)	NA

• all the information are collected at the moment of the cases diagnosed and controls recruited.

In addition, the HapMap Project Phases II and III were also applied in the study, combining them with the ChinaPCA. Considering the close affinity between similar genes, we selected 60 unrelated samples for our analysis for the CEU and YRI. Meanwhile, the CHB (Han Chinese from Beijing) and JPT (Japanese from Tokyo) were pooled with 89 non-affinity Asian individuals, referred to as the ASN. In this study, the numbers of SNPs in each population were 791,208 (ASN), 849,575 (CEU), and 885,926 (YRI) [21]–[23].

### Associations between gene polymorphisms of the RTKs-ERK pathway and prostate cancer

As an important pathway in the development of cancer, the RTKs-ERK pathway could easily induce cancer when its proteins become mutated. Many genes are known to be involved in this pathway, with some related ones as yet undiscovered. In our study, we included almost all the genes known in the pathway for analysis, comprising 40 genes: EGF, EGFR, PDGF, VEGF, MAP2K1, CCND1, and others located from every chromosome. In order to ensure all the SNPs in the pathway were included, we amplified the length of the genes in both directions for 100 kb. Finally, nearly 50,000 SNPs were involved. With these SNPs, systematic association tests for the risk of PCa were conducted, including single-SNP association tests and a gene-based pathway association test. A logistic regression model implemented in Plink [24] was applied to test the associations between the selective SNP polymorphisms and PCa with the covariate of age.

In the gene-based pathway test, the adaptive rank truncated product (ARTP) method was applied. ARTP method was proposed by Yu K et al. [25], and the method was based on the adaptive rank truncated product statistic to combine evidence of associations over different SNPs and genes within a biological pathway. It mainly could be divided into two steps: first, the association evidence between a gene and the outcome was obtained with the standardized summary; second, combine these gene-level P-values into a test statistic for the disease-pathway association. As a powerful statistical method, ARTP could remedy the shortcomings of the rank truncated product (RTP) method, in which the product of K most significant P-values were used as the summary statistic [26]. According to the method, the potential significant association between the genes, pathway, and related diseases could be revealed. In this study, the SNPs with the significant association (*P*<0.05) were collected in the gene-based ARTP analysis. In addition, 10,000 permutations were included in the analysis in order to make the statistics more convincing.

### Natural positive selection loci and eQTL analysis

Although the GWA study indicated that a number of SNPs were associated with prostate cancer, an area analysis of the haplotype could not be included, which might neglect possible related loci. However, the interactions in terms of the functional consequences and evolutionary history of these loci remain largely unknown. Recently, several studies have proposed natural positive selection based on haplotype analysis in various populations [27]–[28]. Candidate regions that are experiencing positive selection could be identified by a number of sophisticated statistical methods. In this study, we applied the integrated haplotype score (iHs), which was developed in 2006 [23], [29]. This test statistic was said to select potential positive selection loci with an empirical threshold of 2 or 1.65. iHS score is derived from the extended haplotype homozygosity statistic (EHH) [30], which measures the decay of the identity of haplotypes that carry a specified “core” allele at one end. It is said that when an allele rises rapidly in frequency due to strong selection, it tends to have high levels of haplotype homozygosity, extending much further than expected under a neutral model. Combining with the EHH, Voight et al. [23] proposed the integrated EHH (iHH), which is divided into iHH_A_ and iHH_B_ with the limitation of computing with respect to an ancestral or derived core allele. In addition, considering the influence of allele frequency on the core SNP, Voight et al. improved the data analysis. And iHS score was conducted with all SNPs with minor allele frequency >5% that were treated as core SNPs. As to the results, both extremely positive and extremely negative iHS scores would be potentially interesting.

In our study, the web-based tool Haplotter [30] was applied to identify iHS scores from the HapMap phase II for three different populations (ASN, CEU and YRI). Considering the various physical locations in the different databases, the number of rs for all the SNP in the RTKs-ERK pathway was selected. According to these findings, iHs values derived with the HapMap Project phase II were confirmed. In addition, Plink was used to explore the association between the ChinaPCA and matched genotypes in all the samples in the pathway. A logistic regression model was used with the PSA value and the covariate was age. On the basis of the SNP (rs), the iHS scores and P-values for the associations were matched. The SNPs, which were said to be significant associations, were collected. Meanwhile, considering the robustness of the selection signals, we continued to conduct a gene-based approach. In this analysis, a window of 50 SNPs centered on each gene closest to the index SNP was created. The candidate targets of selection were defined to be in the upper 10% of the empirical distribution for number of significant SNPs.

When it came to the expression quantitative trait loci (eQTLs) analysis, not only cis- but tran-, the left SNPs with |iHs|>2 were included. Single cis-eQTL considered all association signals from SNPs within 1 Mb up- and down-stream, and trans-eQTLs were identified as being associated with SNPs located greater than 1 Mb from the probe set. The correlation with nearby gene expression was available in the eQTL database Genevar as measured by probes (http://www.sanger.ac.uk/resources/software/genevar/). The HapMap III with 726 individuals (CEU, CHB, GIH, JPT, LWK, MEX, MKK, and YRI), Geneva with 75 individuals (three types of samples: fibroblast, lymphoblastoid cell line, and T cell) and MuTHER healthy female twins with three tissue types (166 adipose, 156 lymphoblastoid cell line, and 160 skin) were collected in this analysis. A linear regression model was used take the distribution of normalized expression levels between genotypes into consideration. And a significance threshold of *P*<0.05 with 10,000-fold permutations was applied to avoid false positive associations.

All analyses were performed using Statistical Analysis System (SAS) software (version 9.0; SAS Institute, Cary, NC, USA) and Plink. All statistical tests were two sided.

## Results

As a key pathway in carcinogenesis, the RTKs-ERK pathway could influence the cell, tissue, and organism directly by mutations in its proteins, which would block normal signal transmission from the extracellular milieu to the DNA. In recent years, many studies have focused on this pathway for the pathogenesis and treatment of diseases, particularly cancers. On the basis of the genes and SNPs of this pathway, the study was conducted with the positive selection loci and eQTL analysis involved.

### SNP-level association and gene-based rank truncated product (ARTP)

We tested the association between each SNP and PCa risk using a logistic regression model after adjusting for age (Table S1). The results showed that 317 loci reached the threshold value of significance (*P*<0.05). When it came to the genes in the RTKs-ERK pathway, three SNPs (rs1815009, rs3743250, and rs3743249) in IGF1R presented with ideal *P*-values (2.84×10^−4^, 2.98×10^−4^, and 4.35×10^−4^), which were in the exon in utr-variant-3-prime. In addition, ERBB2 with six loci (rs2517959, rs2643194, rs2517960, rs2088126, rs903506, and rs2643195) and CCND3 with three loci (rs115597780, rs149917140, and rs4331978) were said to be significant in PCa risk considering their *P*-values.

After the SNP-level analysis, we tested the pathway on the basis of the gene-level ARTP method. Almost all the genes involved in the pathway were selected, with 4276 SNPs reaching significance after the logistic regression model (*P*<0.05) with 10,000 permutations. The gene-based *P*-values derived from ARTP methods were significant for genes ERBB2 (*P* = 0.0034), PTK2 (*P* = 0.0043), FLT1 (*P* = 0.0251), RAF1 (*P* = 0.0173), BIRC2 (*P* = 0.0089), KDR (*P* = 0.0477), and CCND1 (*P* = 0.0394). Meanwhile, the pathway-level test statistic was statistically significant with a *P*-value of 0.0065 (Table 2).

**Table 2:** Results of gene-based pathway associated analysis with Adaptive rank truncated product (ARTP) method.

Genes	*P*	Pathway *P*
IGF1R	0.05919	**0.00660**
**ERBB2**	**0.00340**	
**PTK2**	**0.00430**	
CCND3	0.12108	
**FLT1**	**0.02510**	
EGFR	0.12999	
PDGFD	0.95091	
**RAF1**	**0.01740**	
MAPK3	0.18758	
**BIRC2**	**0.00890**	
**KDR**	**0.04770**	
**CCND1**	**0.03940**	
CCND2	0.58054	
SOS1	0.22248	
PDGFC	0.20288	
VEGFA	0.4960	
EGF	0.56194	
MAP2K1	0.33257	
FLT4	0.43616	
MET	0.12829	
BCL2	0.84392	
IGF1	0.48125	
MAPK1	0.97740	
PDGFB	0.51325	
HGF	0.15429	
FYN	0.95261	
BRAF	0.37986	
GRB2	0.83362	
VEGFC	0.44556	
SOS2	0.64114	
BAD	0.36066	
PDGFRB	0.79392	
PDGFRA	0.67403	
HRAS	0.37000	
VEGFB	0.22218	
XIAP	0.23878	
SHC1	0.56594	
BIRC3	0.75293	
ELK1	0.58034	

• all the results were calculated by ARTP method with the significant SNPs (*P*<0.05) in the loci-level associated analysis collected.

• the significant *P* values were marked in the bold.

### Natural positive selection loci and related eQTL

On the basis of the web-based tool Haplotter, iHs values for all three different ethnic groups (ASN, CEU, and YRI) in HapMap phase II were obtained. Considering our Asian samples, iHs values in ASN were selected according to the number of SNP in the two data sets. In order to analyze the data comprehensively, we defined the threshold as 0.05. In addition, |iHs|>1.65 was used in the next selection. Then, 26 SNPs came into view, which were mainly focused on two genes, EGFR (in the pathway) and MKRN2, located on chromosomes 7 and 16, respectively (Table 3). The maximum values of |iHs| reached 2.967 for rs17172432 (EGFR) with cis-eQTL (*P* = 0.0424, JPT). In addition, the results suggested the association with PCa risk was mainly focused on EGFR. After selecting the potential natural selection loci, we identified the genes with selection in Table 4. The genes were as follows: IGF1 (CEU), EGFR (ASN), ERBB2 (YRI), SHC1 (ASN) GRB2 (ASN and CEU), RAF1 (ASN), MAP2K1 (YRI), PTK2 (CEU), and PDGFB (ASN). With these haplotypes and gene analyses on the positive selection loci, the EGFR was identified again, so we suggested the gene might potentially play an important role in the development and progression of PCa.

**Table 3:** The SNPs which were identified to be significances (*P*<0.05) in the loci-level associated analysis and under nature positive selection (|iHS|>1.65).

CHR	SNP	Gene	OR	L95	U95	P	iHs_ASN	iHs_CEU	iHs_YRI	Gene_ASN	Gene_CEU	Gene_YRI
7	rs11238349	EGFR	0.766	0.6078	0.9653	0.02387	−2.751	−1.532	NA	**+**		
7	rs2302535	EGFR	0.7708	0.6121	0.9707	0.02693	−2.725	−1.503	NA	**+**		
7	rs12535578	EGFR	0.7809	0.6206	0.9826	0.03486	−2.723	−1.734	NA	**+**		
7	rs3800827	EGFR	0.801	0.6543	0.9806	0.03158	−2.538	−1.594	NA	**+**		
7	rs984654	EGFR	0.8001	0.6579	0.9731	0.02558	−2.530	−1.498	NA	**+**		
7	rs3735064	EGFR	0.7874	0.6429	0.9642	0.02077	−2.498	−1.635	−1.003	**+**		
7	rs9642564	EGFR	0.8008	0.6535	0.9814	0.03226	−2.498	−1.635	NA	**+**		
7	rs11766798	EGFR	0.8016	0.6539	0.9826	0.0333	−2.495	−0.906	NA	**+**		
7	rs6978771	EGFR	0.793	0.6476	0.971	0.02475	−2.429	−1.804	NA	**+**		
7	rs11773818	EGFR	0.8013	0.6663	0.9636	0.01856	−2.171	−0.908	NA	**+**		
3	rs9818046	NA	0.7656	0.6098	0.9612	0.0214	−1.675	−0.236	0.343	**+**		
7	rs3735062	EGFR	0.6426	0.4156	0.9934	0.04664	−1.653	NA	NA	**+**		
7	rs17172432	EGFR	0.673	0.525	0.8626	0.001768	2.967	−0.416	0.457	**+**		
7	rs17172434	EGFR	0.6899	0.5297	0.8984	0.005871	2.850	NA	0.020	**+**		
15	rs6494584	NA	0.8549	0.7465	0.979	0.02338	2.233	NA	−0.050			**+**
11	rs7115260	NA	0.1387	0.02398	0.8024	0.0274	2.161	1.318	1.331			
3	rs2255648	MKRN2	0.7628	0.6026	0.9655	0.02431	2.104	1.158	0.365	**+**		
3	rs1542848	MKRN2	0.6941	0.5314	0.9068	0.007408	2.008	0.323	−0.056	**+**		
7	rs17172438	EGFR	0.6003	0.4153	0.8678	0.006644	1.912	−0.424	−0.453	**+**		
7	rs17172436	EGFR	0.6598	0.4401	0.989	0.04405	1.912	NA	0.769	**+**		
7	rs3735063	EGFR	0.638	0.4246	0.9586	0.0305	1.908	NA	NA	**+**		
3	rs2633442	MKRN2	0.7542	0.5822	0.9771	0.03272	1.893	0.850	−0.811	**+**		
3	rs2442802	MKRN2	0.7669	0.5926	0.9925	0.04365	1.893	0.850	−0.811	**+**		
7	rs10488140	EGFR	0.7337	0.5399	0.997	0.04782	1.824	−0.463	−0.139	**+**		
16	rs2005219	BOLA2	0.7858	0.6737	0.9166	0.002144	1.750	0.874	0.211			
3	rs2454431	NA	0.743	0.5746	0.9608	0.02354	1.722	−0.017	−1.581	**+**		
7	rs6958497	EGFR	2.64	1.119	6.227	0.02663	1.647	NA	0.962	**+**		

• the “+” stands for the genes under positive selection.

**Table 4:** The condition of genes in the RTK/ERK pathway under positive selection among three different populations.

Gene	CEU	YRI	ASN
PDGFC	0.12713	0.643733	0.114897
PDGFD	0.257203	0.406681	0.753926
IGF1	**0.067438**	0.54597	0.503402
HGF	0.448157	0.273572	0.420436
EGFR	0.527316	0.889825	**0.07925**
PDGFRA	0.633894	0.889825	0.60804
ERBB2	0.633894	0.040607	0.60804
IGF1R	0.632913	0.755287	0.607936
PDGFRB	0.448157	0.761058	0.361076
FLT1	0.62966	0.641741	0.271252
FLT4	0.12713	0.889825	0.755444
KDR	0.787049	0.761058	0.755444
MET	0.633894	0.185004	0.755444
SHC1	0.633894	0.35688	**0.045331**
GRB2	**0.092378**	0.240116	**0.014238**
SOS1	0.258804	0.643733	0.503402
HRAS	0.787049	0.35688	0.755444
RAF1	0.633894	0.273572	**0.040149**
MAP2K1	0.528917	**0.065686**	0.420436
BAD	0.187752	0.273572	0.420436
BCL2	0.231643	0.753039	0.270781
MAPK3	0.633894	0.761058	0.420436
MAPK1	0.633894	0.13459	0.60804
ELK1	NA	NA	NA
BRAF	0.386864	0.209827	0.755444
PTK2	**0.008314**	0.27204	0.755444
FYN	0.210111	0.207937	0.60626
SOS2	0.633894	0.312085	0.60804
CCND1	0.386864	0.761058	0.214353
CCND2	0.633894	0.643733	0.503402
CCND3	0.528917	0.761058	0.114897
BIRC2	0.528917	0.406732	0.60804
BIRC3	0.528917	0.406732	0.60804
XIAP	NA	NA	NA
EGF	0.12713	0.273572	0.138348
PDGFA	NA	NA	NA
PDGFB	0.633894	0.406732	**0.03816**
VEGFA	0.633894	0.468536	0.755444
VEGFB	0.169059	0.35688	0.420436
VEGFC	0.448157	0.761058	0.19174

• On the basis of the haplotter the positive selections were estimated for almost all the genes in the pathway in which *P*<0.1 was looked as the threshold.

• the significant *P* values were marked in the bold.

eQTL analysis was conducted with the proposed SNPs according to the Sanger method based on three different databases: HapMapIII, MuTHER healthy female twins, and Geneva GenCord individuals. The positive results are presented in Table S2, in which the cis-eQTL and tran-eQTL with the related genes are offered. Significant associations were collected. The results indicated additional SNPs in the pathway might have both cis-regulation and trans-regulation in the genes themselves and nearby (rs11238349, rs17172438, rs984654, rs11773818, and rs17172432) (Table 5). However, the functions could not be repeated in all the HapMap samples derived from similar lymphocytes, different cell types or twins, which suggested specific influences in the different samples.

**Table 5:** The characteristics of the positive loci in our studies with different analysis.

SNP	Gene	Allele	Genotype			loci_iHS	gene_iHS	loci_based *P*	gene_based *P*	cis-eQTL
			mm	Mm	MM					
rs11238349	EGFR	A/G	21	362	1834	−2.751	0.07925	0.02387	0.12999	0.013(CEU)
rs17172438	EGFR	T/C	1	151	2273	1.912	0.07925	0.006644	0.12999	0.0382(YRI)
rs984654	EGFR	C/T	36	540	1848	−2.530	0.07925	0.02558	0.12999	10-4(LCL)
rs11773818	EGFR	T/C	45	620	1759	−2.171	0.07925	0.01856	0.12999	0.0675(fibroblast)/<10^−4^(LCL)
rs17172432	EGFR	T/C	9	353	1991	2.967	0.07925	0.001768	0.12999	0.0424(JPT)

• m, minor allele; M, major allele.

• the iHS values are referred to be the ASN population.

• the eQTL is based on the Sanger with three different databases: HapMapIII, MuTHER healthy female twins and Geneva GenCord individuals.

## Discussion

### Description

PCa is a worldwide disease that causes enormous losses each year considering its high morbidity and mortality. Recently, many studies have been focused on the function of regulatory pathways in the development and progression of related diseases, especially for cancers, [31]–[32], which could provide a way for targeted therapy [33]–[34]. In this study, we proposed that the RTK/ERK pathway could influence various cancers, including PCa, when the relevant proteins are mutated. On the basis of this hypothesis, the association between this pathway and PCa risk was analyzed. Combined with positive selection, the eQTL method, SNP-level association analysis, and a gene-based pathway analysis, we described the essential status of the pathway with regard to PCa. Our results suggest the RTK/ERK pathway has the potential to be a key factor regulating the development and progression of PCa, as we had determined before [17]. According to the analysis we conducted, many genes were said to be significant associated with PCa risk. After combining all the genes we proposed 4 genes (EGFR, ERBB2, PTK2, and RAF1) with five SNPs (rs11238349, rs17172438, rs984654, rs11773818, and rs17172432) as the key factors influencing PCa. In this study, it is suggested many genes in the pathway are significant, especially EGFR, ERBB2, PTK2, and RAF1, which were not only associated with PCa risk, but also under natural selection. This study was based on the natural selection theory to discover potential positive selection in the pathway associated with PCa risk and gave us new insights into studying the relationship between this pathway and the disease.

### Loci-genes analysis and prostate cancer

As one of the significant genes, EGFR was proven to be important in PCa risk. The gene located on Chr7p12 was also determined to be ERBB1, a member of the HER/ERBB/EGFR family of receptors. Recent studies had revealed its close relationship with cancers [35]–[36]. Through both homo- and heterodimeric HER complexes, cell proliferation, motility, and invasion were induced. In addition, it was revealed that obstructing expression and activity of HER-family members could prevent human neoplasia [37]. Therefore, specific anti-EGFR monoclonal antibodies could inhibit cell growth and downregulate the related genes, which could reduce the development of PCa [38]. As the other member of the family, ERBB2, a known proto-oncogene located on the long arm of human chromosome 17 (17q12), is overexpressed in cancers such as breast cancer, with approximately 30% amplification or over-expression of the gene. The gene overexpression could drive aggressive disease, representing a potential therapeutic target [39]–[40]. Recent studies have suggested that EGFR or ERBB2 contribute to prostate cancer (PCa) progression by activating the androgen receptor (AR) under hormone-poor conditions [41]. In 2001, Chen L et al. [42] suggested that dual EGFR/HER2 inhibition combined with androgen withdrawal therapy could sensitize prostate cancer cells to apoptosis. In addition, PTK2 was also suggested to be associated with cancer development. Known as focal adhesion kinase (FAK), PTK2 was a focal adhesion-associated protein kinase involved in cellular adhesion. Lacoste J et al. [43] presented FAK as necessary for the cell motility of PCa and enhancing metastasis. Sumitomo M et al. [44] indicated neutral endopeptidase (NEP) could inhibit FAK phosphorylation on tyrosine, which contributes to the invasion and metastases in PC cells through multiple pathways. In addition, we proposed in the analysis that a well-known proto-oncogene, RAF1, might play a key role in carcinogenesis among humans [45]–[46]. It is a member of the Raf kinase family of serine/threonine-specific protein kinases, in which its brethren kinase B-Raf was the major player in carcinogenesis in humans. Approximately 20% of all examined human tumor samples display a mutated B-Raf gene [47]. Recently, Ren et al. [48] suggested the RAF gene might be the main contributor in the activation of the RAS/RAF/MEK/ERK pathway in Chinese PCa. Keller et al. [49] and Escara-Wilke et al. [50] said Raf kinase inhibitory protein (RKIP) could be a promising factor in therapy. In summary, we concluded from our analysis that four genes (EGFR, ERBB2, PTK2 and RAF1) undergoing positive selection might be important in the regulation of PCa development via various mechanisms. Combined with the close relationship between PCa and these genes, we suggested that these four genes might be key in the RTK/ERK pathway. Through adhesion, cell signaling transduction, regulation of related genes, and assisting gene-gene or protein-protein interactions, the development, progression, and prognosis of the cancers could be regulated. The four genes might be the most promising targets for therapy in the treatment of PCa, especially by virtue of their combined effects.

In this analysis, the four genes (EGFR, ERBB2, PTK2, and RAF1) we proposed were under natural selection, which supported the views we presented above. There are three different types of natural selections: positive selection, negative selection, and balancing selection. Different types of selection would guide the development or purification of the species, in which either positive or negative selection could be more important. At the level of loci and genes, we presented the conditions of evolution for the RTK/ERK pathway. According to their suggestive thresholds (|iHS|>1.65), positive loci were selected. After combining the associated analysis and the eQTL method, five SNPs (rs11238349, rs17172438, rs984654, rs11773818, and rs17172432) were identified as the significant loci that might play a key role in the Pca risk. Tracking recent research, these loci were said to be the possible key factors in the cancer risk. In 2009, Dong et al. [51] studied the role of growth, differentiation, and apoptosis genes in regulating the renal cancer, which implied the rs11238349 in the EGFR might be statistically significantly associated with risk of renal cancer. At the same time, Hong et al. [52] presented the potential significant association in the loci of EGFR (rs11773818 and rs17172432) and breast cancer risk, although without replication, in their stage II analysis. Considering the relationship of these SNPs with other cancers and the function of angiogenesis for the EGFR gene, we inferred that the SNPs we proposed might be associated with the metastasis of Pca to the kidney, breast and other viscera.

With the natural selection effects, the four genes seemed to be more important in regulating the development and progression of Pca based on human population analysis using linkage disequilibrium (LD)-based methods. Explanations for the development of PCa might be associated with evolution at the gene- and locus-level. In addition, combined with the gene-based pathway analysis and molecular evolution method, we did not only explain the key role of the pathway in the development of PCa, but also gained new insight into the potential role of molecular evolution in the pathway.

### Limitation

The presented study combining various analyses has some limitations. First, there are a lot of criteria to evaluate natural selection in a population. As just one of these measurements, iHs might be limited in the estimation. Second, the eQTL and positive selection were based on the available databases, which made it difficult to avoid deficiencies in the results. Finally, every statistical analysis should confirm the deviations. So the real association needs further research.

## Conclusions

In conclusion, prostate cancer is a worldwide disease that brings about inestimable losses for health and the economy. The RTKs-ERK pathway was said to be an important link in this cancer's development and progression. Combining natural selection, gene-based pathway analysis, and other associated analysis, we proposed that the pathway was significantly associated with PCa risk, in which the mutation of EGFR, ERBB2, PTK2, RAF1, and related SNPs in the genes might be the key link in the evolution of the cancer. However, given that the specific locus in the EGFR with positive selection was located in a non-functional intron, the real function of this intron in the evolution of cancers requires further attention.

## Supporting Information

Table S1 **The results of association analysis for Pca risk on the SNPs-level with logistic regression model adjusting for age.**
(DOC)

Table S2 **Expression of quantitative trait loci (eQTLs) analysis with Geneva in three different databases (HapMapIII, MuTHER healthy female twins and Geneva GenCord individuals).** *the results presented in the tables were significant collecting from the total eQTLs analysis for all SNPs in table 3.
(DOC)
