# Supplementary material for: RTK/ERK Pathway under Natural Selection Associated with Prostate Cancer
Source: PLoS One. 2013 Nov 4;8(11):e78254. doi: 10.1371/journal.pone.0078254 (PMC3817240; doi:10.1371/journal.pone.0078254)
Supplement: Table S1 — The results of association analysis for Pca risk on the SNPs-level with logistic regression model adjusting for age. (DOC) [file pone.0078254.s001.doc]

**Table S1 the results of association analysis for Pca risk on the SNPs-level with logistic regression model adjusting for age**

| CHR | SNP | BP | A1 | TEST | NMISS | OR | SE | L95 | U95 | STAT | P |
| --- | --- | --- | --- | --- | --- | --- | --- | --- | --- | --- | --- |
| 15 | rs1815009 | 99504671 | C | ADD | 2389 | 0.7823 | 0.06763 | 0.6852 | 0.8932 | -3.63 | 0.000284 |
| 15 | rs3743250 | 99505316 | T | ADD | 2338 | 0.7815 | 0.06818 | 0.6837 | 0.8932 | -3.617 | 0.000298 |
| 15 | rs3743249 | 99505423 | T | ADD | 2389 | 0.7885 | 0.06757 | 0.6907 | 0.9001 | -3.518 | 0.000435 |
| 17 | rs2517959 | 37846512 | T | ADD | 2368 | 0.7866 | 0.06924 | 0.6868 | 0.9009 | -3.467 | 0.000527 |
| 17 | rs2643194 | 37853048 | T | ADD | 1959 | 0.7668 | 0.07685 | 0.6596 | 0.8914 | -3.455 | 0.00055 |
| 17 | rs2517960 | 37846521 | C | ADD | 2374 | 0.7874 | 0.06919 | 0.6876 | 0.9018 | -3.454 | 0.000552 |
| 8 | rs138246471 | 1.42E+08 | G | ADD | 2343 | 0.2969 | 0.3553 | 0.148 | 0.5957 | -3.418 | 0.000631 |
| 6 | rs115597780 | 42007877 | T | ADD | 1780 | 0.3681 | 0.2952 | 0.2064 | 0.6564 | -3.386 | 0.000708 |
| 6 | rs149917140 | 42010175 | T | ADD | 1808 | 0.3815 | 0.2854 | 0.2181 | 0.6674 | -3.377 | 0.000733 |
| 6 | rs4331978 | 42009331 | T | ADD | 1797 | 0.3762 | 0.2896 | 0.2133 | 0.6636 | -3.376 | 0.000735 |
| 17 | rs2088126 | 37879030 | G | ADD | 1907 | 0.7697 | 0.07755 | 0.6611 | 0.896 | -3.376 | 0.000736 |
| 17 | rs903506 | 37879762 | A | ADD | 1900 | 0.7691 | 0.07788 | 0.6603 | 0.896 | -3.37 | 0.000751 |
| 17 | rs2643195 | 37853118 | G | ADD | 1960 | 0.7736 | 0.07674 | 0.6656 | 0.8992 | -3.345 | 0.000823 |
| 17 | rs4252627 | 37868715 | T | ADD | 1906 | 0.7736 | 0.07815 | 0.6638 | 0.9017 | -3.284 | 0.001023 |
| 6 | rs78790875 | 42009106 | G | ADD | 1792 | 0.3907 | 0.2887 | 0.2219 | 0.6881 | -3.255 | 0.001135 |
| 17 | rs2517951 | 37853097 | T | ADD | 1957 | 0.7788 | 0.07692 | 0.6698 | 0.9055 | -3.25 | 0.001152 |
| 17 | rs2904766 | 37848677 | G | ADD | 1880 | 0.7725 | 0.07968 | 0.6608 | 0.903 | -3.24 | 0.001196 |
| 17 | rs1810132 | 37866005 | T | ADD | 1913 | 0.7776 | 0.07769 | 0.6678 | 0.9055 | -3.237 | 0.001207 |
| 17 | rs2934971 | 37854507 | T | ADD | 1936 | 0.7805 | 0.07726 | 0.6708 | 0.9081 | -3.207 | 0.00134 |
| 15 | rs2654981 | 99505129 | C | ADD | 2155 | 1.264 | 0.07317 | 1.095 | 1.458 | 3.197 | 0.001388 |
| 6 | rs79132119 | 42000303 | T | ADD | 1705 | 0.3667 | 0.3142 | 0.1981 | 0.6787 | -3.193 | 0.001406 |
| 17 | rs61552325 | 37884037 | G | ADD | 1870 | 0.7795 | 0.07831 | 0.6686 | 0.9088 | -3.181 | 0.001465 |
| 17 | rs2934967 | 37870378 | A | ADD | 1901 | 0.7813 | 0.07771 | 0.6709 | 0.9098 | -3.176 | 0.001494 |
| 13 | rs7998124 | 28917308 | T | ADD | 1971 | 0.7341 | 0.0982 | 0.6056 | 0.8899 | -3.147 | 0.001648 |
| 17 | rs2952156 | 37876835 | G | ADD | 1896 | 0.7828 | 0.07787 | 0.672 | 0.9119 | -3.144 | 0.001667 |
| 7 | rs17172432 | 55141317 | C | ADD | 2321 | 0.673 | 0.1267 | 0.525 | 0.8626 | -3.127 | 0.001768 |
| 6 | rs80080085 | 41999003 | T | ADD | 1701 | 0.3765 | 0.317 | 0.2023 | 0.7009 | -3.081 | 0.002063 |
| 17 | rs1565923 | 37858678 | G | ADD | 1922 | 0.7887 | 0.07741 | 0.6777 | 0.9179 | -3.067 | 0.002165 |
| 17 | rs2952157 | 37877412 | A | ADD | 1885 | 0.7887 | 0.07826 | 0.6765 | 0.9194 | -3.033 | 0.002419 |
| 6 | rs144352608 | 42001948 | T | ADD | 1626 | 0.3961 | 0.3058 | 0.2176 | 0.7213 | -3.028 | 0.002459 |
| 11 | rs191482937 | 1.04E+08 | G | ADD | 1417 | 0.4369 | 0.274 | 0.2554 | 0.7475 | -3.022 | 0.002512 |
| 6 | rs74916404 | 42012488 | T | ADD | 1858 | 0.4636 | 0.2577 | 0.2798 | 0.7683 | -2.982 | 0.00286 |
| 17 | rs2952155 | 37861718 | C | ADD | 1916 | 0.7943 | 0.07721 | 0.6828 | 0.9241 | -2.982 | 0.002861 |
| 17 | rs11653998 | 37877447 | G | ADD | 1881 | 0.7929 | 0.07829 | 0.6801 | 0.9244 | -2.965 | 0.003031 |
| 6 | rs147804102 | 42004869 | T | ADD | 1741 | 0.3947 | 0.3145 | 0.2131 | 0.7311 | -2.956 | 0.003121 |
| 15 | rs8038415 | 99499434 | T | ADD | 2134 | 1.235 | 0.07137 | 1.074 | 1.42 | 2.955 | 0.003128 |
| 15 | rs2016347 | 99503800 | G | ADD | 2329 | 0.8188 | 0.06879 | 0.7155 | 0.937 | -2.906 | 0.003659 |
| 3 | rs13086363 | 12675709 | T | ADD | 2369 | 0.6767 | 0.1346 | 0.5198 | 0.8811 | -2.9 | 0.003728 |
| 15 | rs8033670 | 99498879 | T | ADD | 2230 | 1.224 | 0.06966 | 1.068 | 1.403 | 2.899 | 0.003738 |
| 16 | rs28529403 | 30134656 | T | ADD | 1466 | 0.787 | 0.08444 | 0.667 | 0.9287 | -2.836 | 0.004563 |
| 15 | rs2684788 | 99504437 | T | ADD | 2389 | 0.8251 | 0.06787 | 0.7224 | 0.9425 | -2.832 | 0.004625 |
| 11 | rs10895290 | 1.02E+08 | A | ADD | 2390 | 0.7987 | 0.07941 | 0.6836 | 0.9332 | -2.831 | 0.004645 |
| 6 | rs148447776 | 42001134 | A | ADD | 1707 | 0.4228 | 0.3067 | 0.2318 | 0.7712 | -2.807 | 0.004997 |
| 3 | rs12634077 | 12674566 | A | ADD | 2371 | 0.6862 | 0.1341 | 0.5276 | 0.8926 | -2.807 | 0.004998 |
| 7 | rs74581689 | 55102337 | T | ADD | 2256 | 0.517 | 0.2361 | 0.3255 | 0.8213 | -2.794 | 0.005207 |
| 3 | rs1391868 | 12679466 | T | ADD | 2371 | 0.6884 | 0.1342 | 0.5292 | 0.8957 | -2.781 | 0.005421 |
| 15 | rs2872060 | 99499493 | T | ADD | 2141 | 1.218 | 0.07108 | 1.059 | 1.4 | 2.772 | 0.005563 |
| 3 | rs78815931 | 12678203 | G | ADD | 2372 | 0.6895 | 0.1342 | 0.53 | 0.8969 | -2.771 | 0.005584 |
| 7 | rs17172434 | 55144430 | G | ADD | 2284 | 0.6899 | 0.1348 | 0.5297 | 0.8984 | -2.755 | 0.005871 |
| 6 | rs147577568 | 42001911 | A | ADD | 1690 | 0.4275 | 0.3094 | 0.2331 | 0.7838 | -2.747 | 0.006007 |
| 4 | rs55916282 | 55993306 | A | ADD | 2331 | 1.352 | 0.1104 | 1.089 | 1.679 | 2.734 | 0.006255 |
| 7 | rs17172438 | 55151537 | C | ADD | 2390 | 0.6003 | 0.188 | 0.4153 | 0.8678 | -2.714 | 0.006644 |
| 3 | rs4684867 | 12659494 | G | ADD | 2371 | 0.6964 | 0.1345 | 0.535 | 0.9064 | -2.691 | 0.007133 |
| 15 | rs9672254 | 99498085 | C | ADD | 2217 | 1.207 | 0.07033 | 1.052 | 1.386 | 2.679 | 0.007374 |
| 13 | rs622227 | 29039214 | C | ADD | 2390 | 1.532 | 0.1629 | 1.113 | 2.108 | 2.616 | 0.008899 |
| 3 | rs5746207 | 12648156 | G | ADD | 2355 | 0.6994 | 0.1368 | 0.5349 | 0.9144 | -2.614 | 0.008937 |
| 11 | rs78968063 | 69457069 | A | ADD | 1949 | 0.04467 | 1.191 | 0.004331 | 0.4606 | -2.611 | 0.009026 |
| 7 | rs12538371 | 55236020 | C | ADD | 2337 | 0.1536 | 0.718 | 0.03759 | 0.6273 | -2.61 | 0.009067 |
| 6 | rs142937763 | 42012786 | T | ADD | 1915 | 0.4891 | 0.2751 | 0.2853 | 0.8387 | -2.6 | 0.009335 |
| 7 | rs66740303 | 55159875 | C | ADD | 2200 | 0.5336 | 0.2417 | 0.3323 | 0.8569 | -2.599 | 0.009352 |
| 16 | rs61764202 | 30134679 | C | ADD | 1579 | 0.8097 | 0.08132 | 0.6904 | 0.9496 | -2.595 | 0.009452 |
| 12 | rs3217805 | 4388084 | G | ADD | 1268 | 0.3273 | 0.431 | 0.1406 | 0.7617 | -2.592 | 0.009554 |
| 17 | rs2904767 | 37850541 | C | ADD | 933 | 0.6597 | 0.1605 | 0.4816 | 0.9037 | -2.591 | 0.009577 |
| 3 | rs5746194 | 12658520 | G | ADD | 2158 | 0.7044 | 0.1354 | 0.5402 | 0.9186 | -2.587 | 0.00968 |
| 3 | rs13069889 | 12667680 | T | ADD | 2312 | 0.7056 | 0.1352 | 0.5413 | 0.9197 | -2.579 | 0.00992 |
| 3 | rs904464 | 12642949 | C | ADD | 2372 | 0.7108 | 0.1328 | 0.5479 | 0.9221 | -2.57 | 0.01016 |
| 2 | rs56054777 | 39278250 | G | ADD | 2296 | 0.2798 | 0.4961 | 0.1058 | 0.7397 | -2.568 | 0.01023 |
| 3 | rs12629082 | 12669448 | G | ADD | 2156 | 0.7014 | 0.1381 | 0.535 | 0.9194 | -2.568 | 0.01023 |
| 15 | rs2871866 | 99221888 | C | ADD | 1859 | 0.8198 | 0.07741 | 0.7044 | 0.9541 | -2.566 | 0.01028 |
| 4 | rs140233478 | 1.58E+08 | T | ADD | 2328 | 4.175 | 0.557 | 1.401 | 12.44 | 2.566 | 0.01029 |
| 4 | rs150753022 | 1.58E+08 | A | ADD | 2328 | 4.169 | 0.5569 | 1.4 | 12.42 | 2.564 | 0.01036 |
| 6 | rs3025012 | 43747962 | G | ADD | 1474 | 0.3158 | 0.4517 | 0.1303 | 0.7654 | -2.552 | 0.01071 |
| 11 | rs9344 | 69462910 | G | ADD | 229 | 1.693 | 0.2068 | 1.129 | 2.539 | 2.547 | 0.01088 |
| 6 | rs3025018 | 43748795 | T | ADD | 758 | 0.3419 | 0.4225 | 0.1494 | 0.7827 | -2.54 | 0.01109 |
| 3 | rs6795441 | 12702562 | A | ADD | 2389 | 0.7327 | 0.1228 | 0.576 | 0.9321 | -2.533 | 0.01131 |
| 4 | rs11569095 | 1.11E+08 | G | ADD | 2377 | 23.92 | 1.254 | 2.048 | 279.3 | 2.532 | 0.01135 |
| 4 | rs187724013 | 1.58E+08 | G | ADD | 2348 | 5.03 | 0.6387 | 1.439 | 17.59 | 2.529 | 0.01143 |
| 15 | rs142022576 | 66713127 | A | ADD | 519 | 2.197 | 0.3113 | 1.194 | 4.044 | 2.529 | 0.01145 |
| 3 | rs11713601 | 12662117 | G | ADD | 2373 | 0.7133 | 0.1337 | 0.5489 | 0.927 | -2.527 | 0.0115 |
| 3 | rs6792773 | 12668726 | T | ADD | 2376 | 0.5746 | 0.2195 | 0.3737 | 0.8836 | -2.523 | 0.01162 |
| 8 | rs145939286 | 1.42E+08 | A | ADD | 2136 | 0.177 | 0.6895 | 0.04581 | 0.6836 | -2.512 | 0.01202 |
| 15 | rs3743251 | 99504129 | A | ADD | 2387 | 0.8425 | 0.06858 | 0.7366 | 0.9637 | -2.499 | 0.01246 |
| 6 | rs7738113 | 41962692 | T | ADD | 2022 | 0.1198 | 0.8522 | 0.02254 | 0.6364 | -2.49 | 0.01276 |
| 7 | rs2286962 | 55114093 | C | ADD | 2323 | 0.6094 | 0.1991 | 0.4125 | 0.9002 | -2.488 | 0.01283 |
| 3 | rs73130368 | 12667374 | T | ADD | 2375 | 0.5795 | 0.2202 | 0.3764 | 0.8922 | -2.478 | 0.0132 |
| 13 | rs7995976 | 28941060 | A | ADD | 2389 | 0.8381 | 0.07167 | 0.7283 | 0.9645 | -2.464 | 0.01375 |
| 3 | rs2348199 | 12691265 | G | ADD | 2365 | 0.7161 | 0.1357 | 0.5488 | 0.9344 | -2.46 | 0.01388 |
| 15 | rs10438491 | 99489265 | T | ADD | 2389 | 0.8484 | 0.06684 | 0.7442 | 0.9672 | -2.46 | 0.01391 |
| 3 | rs2596830 | 12631774 | G | ADD | 2373 | 0.7218 | 0.1331 | 0.5561 | 0.937 | -2.449 | 0.01431 |
| 3 | rs9852359 | 12693430 | T | ADD | 2374 | 0.5829 | 0.2211 | 0.3779 | 0.8991 | -2.441 | 0.01464 |
| 3 | rs4684871 | 12697498 | G | ADD | 2389 | 0.7272 | 0.1311 | 0.5623 | 0.9403 | -2.43 | 0.01512 |
| 3 | rs10460973 | 12704163 | C | ADD | 2372 | 0.7395 | 0.1251 | 0.5787 | 0.9451 | -2.412 | 0.01588 |
| 3 | rs904453 | 12704894 | G | ADD | 2372 | 0.7395 | 0.1251 | 0.5787 | 0.9451 | -2.412 | 0.01588 |
| 4 | rs111260399 | 1.11E+08 | G | ADD | 2318 | 0.3942 | 0.3864 | 0.1849 | 0.8407 | -2.409 | 0.016 |
| 13 | rs625980 | 29038388 | T | ADD | 2373 | 1.495 | 0.1675 | 1.076 | 2.076 | 2.4 | 0.01641 |
| 3 | rs9809947 | 12655944 | C | ADD | 2368 | 0.5748 | 0.2314 | 0.3652 | 0.9047 | -2.393 | 0.01672 |
| 3 | rs2016492 | 12634518 | C | ADD | 2346 | 0.74 | 0.1262 | 0.5779 | 0.9476 | -2.386 | 0.01703 |
| 13 | rs56728557 | 29029431 | A | ADD | 2372 | 1.495 | 0.1684 | 1.074 | 2.079 | 2.386 | 0.01704 |
| 3 | rs5746182 | 12672550 | G | ADD | 2381 | 0.6003 | 0.2143 | 0.3944 | 0.9137 | -2.381 | 0.01726 |
| 3 | rs57884385 | 12673551 | C | ADD | 2381 | 0.6003 | 0.2143 | 0.3944 | 0.9137 | -2.381 | 0.01726 |
| 15 | rs74325933 | 99321452 | C | ADD | 2017 | 0.7383 | 0.1277 | 0.5748 | 0.9483 | -2.376 | 0.01751 |
| 3 | rs77707425 | 12675810 | C | ADD | 2379 | 0.5988 | 0.2161 | 0.3921 | 0.9146 | -2.373 | 0.01764 |
| 13 | rs6491275 | 28941930 | A | ADD | 2376 | 0.8437 | 0.0717 | 0.7331 | 0.971 | -2.371 | 0.01775 |
| 3 | rs111654948 | 12677599 | A | ADD | 2338 | 0.5332 | 0.2661 | 0.3165 | 0.8982 | -2.364 | 0.0181 |
| 5 | rs11249739 | 1.8E+08 | T | ADD | 2166 | 0.8036 | 0.09256 | 0.6703 | 0.9635 | -2.362 | 0.01817 |
| 7 | rs1858830 | 1.16E+08 | C | ADD | 1089 | 1.261 | 0.09838 | 1.04 | 1.529 | 2.358 | 0.01836 |
| 18 | rs145099651 | 60836658 | T | ADD | 1826 | 7.706 | 0.8666 | 1.41 | 42.12 | 2.356 | 0.01846 |
| 7 | rs729969 | 55128207 | A | ADD | 2380 | 0.6379 | 0.1908 | 0.4389 | 0.9273 | -2.356 | 0.01849 |
| 7 | rs11773818 | 55123968 | C | ADD | 2389 | 0.8013 | 0.09411 | 0.6663 | 0.9636 | -2.354 | 0.01856 |
| 3 | rs2454437 | 12638730 | A | ADD | 2375 | 0.7343 | 0.1312 | 0.5677 | 0.9497 | -2.353 | 0.01862 |
| 3 | rs4234513 | 12703063 | G | ADD | 2286 | 0.7452 | 0.1252 | 0.5831 | 0.9525 | -2.349 | 0.01883 |
| 18 | rs1800477 | 60985773 | T | ADD | 1826 | 0.4166 | 0.3741 | 0.2001 | 0.8672 | -2.341 | 0.01925 |
| 7 | rs143393162 | 55105896 | A | ADD | 2333 | 2.803 | 0.441 | 1.181 | 6.654 | 2.337 | 0.01943 |
| 3 | rs11709504 | 12674199 | C | ADD | 2382 | 0.6081 | 0.2134 | 0.4003 | 0.9238 | -2.332 | 0.01973 |
| 5 | rs35006544 | 1.8E+08 | T | ADD | 706 | 0.7484 | 0.1243 | 0.5866 | 0.9549 | -2.331 | 0.01974 |
| 7 | rs2283051 | 1.16E+08 | C | ADD | 2215 | 0.3053 | 0.5096 | 0.1125 | 0.8289 | -2.328 | 0.0199 |
| 3 | rs9849807 | 12675026 | G | ADD | 2380 | 0.6067 | 0.2151 | 0.398 | 0.9249 | -2.323 | 0.02017 |
| 8 | rs9657438 | 1.42E+08 | T | ADD | 743 | 0.1786 | 0.7427 | 0.04165 | 0.7657 | -2.319 | 0.02037 |
| 7 | rs12669701 | 55131670 | A | ADD | 2248 | 0.7831 | 0.1055 | 0.6367 | 0.963 | -2.317 | 0.02051 |
| 7 | rs3735064 | 55144833 | C | ADD | 2348 | 0.7874 | 0.1034 | 0.6429 | 0.9642 | -2.312 | 0.02077 |
| 12 | rs150708329 | 1.03E+08 | G | ADD | 1683 | 0.2639 | 0.5797 | 0.08471 | 0.8219 | -2.298 | 0.02155 |
| 3 | rs111962445 | 12695689 | A | ADD | 2361 | 0.6014 | 0.2219 | 0.3892 | 0.9291 | -2.291 | 0.02194 |
| 4 | rs144522369 | 1.58E+08 | C | ADD | 2044 | 0.2486 | 0.6078 | 0.07554 | 0.8182 | -2.29 | 0.02202 |
| 3 | rs2442807 | 12629965 | C | ADD | 2375 | 0.7403 | 0.1315 | 0.5722 | 0.9579 | -2.287 | 0.02218 |
| 13 | rs7317419 | 29025559 | T | ADD | 2383 | 1.481 | 0.1717 | 1.058 | 2.073 | 2.286 | 0.02223 |
| 13 | rs10507386 | 29028554 | T | ADD | 2389 | 1.47 | 0.1689 | 1.056 | 2.047 | 2.282 | 0.02248 |
| 15 | rs1398873 | 99396105 | T | ADD | 1585 | 0.6099 | 0.2169 | 0.3987 | 0.933 | -2.28 | 0.02262 |
| 11 | rs881365 | 1.04E+08 | T | ADD | 1901 | 0.7121 | 0.1491 | 0.5316 | 0.9538 | -2.277 | 0.02276 |
| 3 | rs9817675 | 12676113 | T | ADD | 2381 | 0.6146 | 0.2141 | 0.404 | 0.9351 | -2.273 | 0.023 |
| 15 | rs7162314 | 99489916 | G | ADD | 2274 | 0.8548 | 0.06903 | 0.7467 | 0.9787 | -2.272 | 0.02309 |
| 13 | rs3794402 | 28924832 | C | ADD | 2298 | 2.008 | 0.3075 | 1.099 | 3.668 | 2.267 | 0.02338 |
| 15 | rs8034284 | 99496248 | C | ADD | 2101 | 1.175 | 0.07123 | 1.022 | 1.351 | 2.266 | 0.02342 |
| 8 | rs76469750 | 1.42E+08 | T | ADD | 2353 | 0.538 | 0.2736 | 0.3147 | 0.9197 | -2.266 | 0.02345 |
| 5 | rs10071147 | 1.8E+08 | A | ADD | 2002 | 1.183 | 0.07419 | 1.023 | 1.368 | 2.265 | 0.02353 |
| 13 | rs619031 | 29039871 | T | ADD | 2352 | 1.504 | 0.1803 | 1.056 | 2.141 | 2.262 | 0.02367 |
| 8 | rs28438589 | 1.42E+08 | C | ADD | 1166 | 0.7181 | 0.1464 | 0.5389 | 0.9568 | -2.262 | 0.02372 |
| 3 | rs7637392 | 12696433 | G | ADD | 2375 | 0.6102 | 0.2185 | 0.3976 | 0.9364 | -2.26 | 0.02379 |
| 7 | rs11238349 | 55156071 | G | ADD | 2185 | 0.766 | 0.118 | 0.6078 | 0.9653 | -2.259 | 0.02387 |
| 8 | rs143510792 | 1.42E+08 | T | ADD | 2327 | 0.2692 | 0.5826 | 0.08592 | 0.8432 | -2.253 | 0.02427 |
| 3 | rs963959 | 12649937 | T | ADD | 2354 | 0.7277 | 0.1412 | 0.5517 | 0.9598 | -2.251 | 0.0244 |
| 5 | rs79234561 | 1.8E+08 | A | ADD | 2125 | 0.1429 | 0.8651 | 0.02622 | 0.7787 | -2.249 | 0.02451 |
| 13 | rs9508017 | 28934364 | T | ADD | 2306 | 0.8497 | 0.07253 | 0.7371 | 0.9795 | -2.246 | 0.02472 |
| 7 | rs6978771 | 55140296 | C | ADD | 2340 | 0.793 | 0.1033 | 0.6476 | 0.971 | -2.245 | 0.02475 |
| 3 | rs59821954 | 12687613 | C | ADD | 2377 | 0.6127 | 0.2185 | 0.3992 | 0.9402 | -2.242 | 0.02495 |
| 15 | rs145732636 | 99452581 | T | ADD | 2154 | 0.5924 | 0.2342 | 0.3743 | 0.9376 | -2.235 | 0.02541 |
| 7 | rs984654 | 55144156 | T | ADD | 2389 | 0.8001 | 0.09988 | 0.6579 | 0.9731 | -2.233 | 0.02558 |
| 16 | rs11865086 | 30130493 | C | ADD | 2387 | 0.855 | 0.07021 | 0.7451 | 0.9811 | -2.232 | 0.02561 |
| 3 | rs11923427 | 12663835 | G | ADD | 2374 | 0.611 | 0.2208 | 0.3964 | 0.9418 | -2.232 | 0.02563 |
| 3 | rs3773345 | 12642945 | C | ADD | 2371 | 0.6073 | 0.2237 | 0.3917 | 0.9416 | -2.229 | 0.02582 |
| 13 | rs6490306 | 29025304 | A | ADD | 2378 | 1.47 | 0.173 | 1.047 | 2.063 | 2.226 | 0.02599 |
| 3 | rs56363132 | 12680403 | C | ADD | 2376 | 0.6145 | 0.2188 | 0.4002 | 0.9435 | -2.226 | 0.02602 |
| 3 | rs9837418 | 12685012 | A | ADD | 2376 | 0.6145 | 0.2188 | 0.4002 | 0.9435 | -2.226 | 0.02602 |
| 7 | rs75546857 | 55114721 | G | ADD | 2328 | 0.6472 | 0.1957 | 0.441 | 0.9498 | -2.223 | 0.02622 |
| 7 | rs6958497 | 55161746 | C | ADD | 1480 | 2.64 | 0.4379 | 1.119 | 6.227 | 2.217 | 0.02663 |
| 11 | rs4385869 | 1.02E+08 | A | ADD | 1109 | 0.7975 | 0.1021 | 0.6529 | 0.9741 | -2.217 | 0.02664 |
| 3 | rs9864781 | 12683446 | T | ADD | 2360 | 0.5925 | 0.2362 | 0.373 | 0.9413 | -2.216 | 0.02669 |
| 7 | rs38846 | 1.16E+08 | C | ADD | 2159 | 0.4244 | 0.3871 | 0.1988 | 0.9063 | -2.214 | 0.02683 |
| 7 | rs2302535 | 55154688 | A | ADD | 2188 | 0.7708 | 0.1177 | 0.6121 | 0.9707 | -2.213 | 0.02693 |
| 5 | rs13165395 | 1.8E+08 | C | ADD | 1563 | 1.269 | 0.1077 | 1.027 | 1.567 | 2.211 | 0.02702 |
| 13 | rs3209052 | 28874695 | C | ADD | 1856 | 0.8146 | 0.09277 | 0.6791 | 0.977 | -2.211 | 0.02707 |
| 3 | rs76427221 | 12646751 | A | ADD | 2253 | 0.5427 | 0.2766 | 0.3156 | 0.9334 | -2.209 | 0.02717 |
| 13 | rs7982257 | 28921481 | G | ADD | 2167 | 0.8451 | 0.07619 | 0.7279 | 0.9812 | -2.208 | 0.02721 |
| 5 | rs3776413 | 1.8E+08 | T | ADD | 1849 | 0.7976 | 0.1024 | 0.6525 | 0.9749 | -2.208 | 0.02723 |
| 3 | rs1874942 | 12690343 | G | ADD | 2154 | 0.7407 | 0.1362 | 0.5672 | 0.9673 | -2.204 | 0.02751 |
| 13 | rs58518391 | 29031060 | A | ADD | 2385 | 1.454 | 0.1702 | 1.042 | 2.03 | 2.2 | 0.02784 |
| 13 | rs58759217 | 29030978 | T | ADD | 2386 | 1.454 | 0.1702 | 1.041 | 2.03 | 2.199 | 0.0279 |
| 6 | rs3025015 | 43748350 | A | ADD | 1782 | 0.4302 | 0.384 | 0.2027 | 0.9131 | -2.197 | 0.02805 |
| 11 | rs183883220 | 1.04E+08 | A | ADD | 2357 | 0.1451 | 0.8808 | 0.02582 | 0.8157 | -2.191 | 0.02843 |
| 11 | rs149330409 | 1.04E+08 | T | ADD | 2357 | 0.1451 | 0.8808 | 0.02582 | 0.8157 | -2.191 | 0.02843 |
| 7 | rs115391410 | 55113796 | C | ADD | 2322 | 0.6422 | 0.2027 | 0.4316 | 0.9554 | -2.185 | 0.02887 |
| 7 | rs11977660 | 55162336 | T | ADD | 2390 | 0.8577 | 0.0703 | 0.7473 | 0.9844 | -2.184 | 0.02899 |
| 7 | rs79868943 | 55159109 | A | ADD | 2360 | 0.6313 | 0.2108 | 0.4176 | 0.9543 | -2.182 | 0.02911 |
| 13 | rs34867831 | 29022711 | C | ADD | 2339 | 1.502 | 0.1864 | 1.042 | 2.164 | 2.182 | 0.02915 |
| 22 | rs2329881 | 22170312 | T | ADD | 1356 | 0.1459 | 0.8838 | 0.02582 | 0.825 | -2.178 | 0.02943 |
| 3 | rs9875104 | 12685243 | T | ADD | 2145 | 0.5643 | 0.2635 | 0.3367 | 0.9458 | -2.171 | 0.02991 |
| 15 | rs2312491 | 99508180 | A | ADD | 1956 | 0.8474 | 0.07625 | 0.7298 | 0.9841 | -2.171 | 0.02994 |
| 13 | rs17626553 | 28968770 | C | ADD | 2367 | 1.908 | 0.2977 | 1.065 | 3.42 | 2.17 | 0.02998 |
| 15 | rs45578135 | 99485516 | C | ADD | 1621 | 0.598 | 0.237 | 0.3758 | 0.9515 | -2.17 | 0.03002 |
| 7 | rs17336017 | 55152680 | A | ADD | 2364 | 0.638 | 0.2077 | 0.4246 | 0.9586 | -2.164 | 0.0305 |
| 7 | rs3735063 | 55153164 | C | ADD | 2364 | 0.638 | 0.2077 | 0.4246 | 0.9586 | -2.164 | 0.0305 |
| 5 | rs77738372 | 1.8E+08 | T | ADD | 2105 | 0.1865 | 0.7763 | 0.04073 | 0.854 | -2.163 | 0.03052 |
| 13 | rs77616576 | 29041593 | T | ADD | 1632 | 0.2186 | 0.7033 | 0.05508 | 0.8676 | -2.162 | 0.03062 |
| 22 | rs6001509 | 39624900 | T | ADD | 1541 | 0.6336 | 0.2111 | 0.4189 | 0.9584 | -2.161 | 0.03066 |
| 4 | rs2298989 | 1.11E+08 | T | ADD | 2388 | 1.167 | 0.07169 | 1.014 | 1.344 | 2.159 | 0.03084 |
| 13 | rs9513085 | 28920923 | C | ADD | 2161 | 0.8484 | 0.07625 | 0.7306 | 0.9851 | -2.157 | 0.03103 |
| 15 | rs12591122 | 99498412 | C | ADD | 2241 | 0.8608 | 0.06954 | 0.7512 | 0.9866 | -2.155 | 0.03119 |
| 13 | rs2093821 | 28969209 | C | ADD | 2382 | 1.753 | 0.2607 | 1.052 | 2.923 | 2.154 | 0.03121 |
| 7 | rs75144654 | 551646 | A | ADD | 207 | 0.1562 | 0.8629 | 0.02878 | 0.8476 | -2.152 | 0.03143 |
| 13 | rs118137198 | 29023288 | C | ADD | 2350 | 1.498 | 0.188 | 1.036 | 2.166 | 2.15 | 0.03153 |
| 7 | rs3800827 | 55142654 | A | ADD | 2348 | 0.801 | 0.1032 | 0.6543 | 0.9806 | -2.15 | 0.03158 |
| 8 | rs80022884 | 1.42E+08 | T | ADD | 2113 | 0.2158 | 0.7149 | 0.05316 | 0.8762 | -2.145 | 0.03197 |
| 17 | rs2904768 | 37850571 | T | ADD | 1493 | 0.8151 | 0.09531 | 0.6762 | 0.9825 | -2.145 | 0.03197 |
| 5 | rs899169 | 1.8E+08 | A | ADD | 2053 | 0.8619 | 0.06935 | 0.7524 | 0.9874 | -2.143 | 0.03215 |
| 7 | rs9642564 | 55145334 | G | ADD | 2342 | 0.8008 | 0.1037 | 0.6535 | 0.9814 | -2.141 | 0.03226 |
| 13 | rs17537653 | 28968510 | A | ADD | 2365 | 1.894 | 0.2983 | 1.056 | 3.398 | 2.141 | 0.03226 |
| 3 | rs75133077 | 12688513 | T | ADD | 2375 | 0.6249 | 0.2198 | 0.4062 | 0.9613 | -2.14 | 0.03238 |
| 3 | rs55762590 | 12689451 | T | ADD | 2375 | 0.6249 | 0.2198 | 0.4062 | 0.9613 | -2.14 | 0.03238 |
| 3 | rs6766666 | 12690855 | T | ADD | 2375 | 0.6249 | 0.2198 | 0.4062 | 0.9613 | -2.14 | 0.03238 |
| 8 | rs150558261 | 1.42E+08 | C | ADD | 2119 | 0.2169 | 0.7143 | 0.05347 | 0.8795 | -2.14 | 0.03238 |
| 16 | rs7542 | 30125840 | G | ADD | 2165 | 0.8544 | 0.07354 | 0.7397 | 0.9869 | -2.14 | 0.03239 |
| 7 | rs79698813 | 55135897 | G | ADD | 2362 | 0.6496 | 0.2017 | 0.4375 | 0.9645 | -2.139 | 0.0324 |
| 15 | rs12437796 | 99493653 | A | ADD | 2262 | 0.8615 | 0.06971 | 0.7514 | 0.9876 | -2.139 | 0.03242 |
| 15 | rs35115159 | 99490912 | G | ADD | 2304 | 0.8625 | 0.06918 | 0.7532 | 0.9878 | -2.138 | 0.03253 |
| 15 | rs12592205 | 99490596 | T | ADD | 2309 | 0.8627 | 0.06911 | 0.7535 | 0.9879 | -2.136 | 0.03266 |
| 13 | rs2256849 | 29045852 | G | ADD | 2279 | 1.45 | 0.1739 | 1.031 | 2.039 | 2.136 | 0.03269 |
| 7 | rs5745765 | 81332213 | C | ADD | 1630 | 0.5199 | 0.3071 | 0.2848 | 0.9491 | -2.13 | 0.03317 |
| 4 | rs148833685 | 1.11E+08 | A | ADD | 2304 | 0.4456 | 0.3796 | 0.2118 | 0.9377 | -2.129 | 0.03322 |
| 18 | rs34971240 | 60923936 | G | ADD | 428 | 0.5595 | 0.2728 | 0.3278 | 0.955 | -2.129 | 0.03328 |
| 7 | rs11766798 | 55124319 | G | ADD | 2334 | 0.8016 | 0.1039 | 0.6539 | 0.9826 | -2.128 | 0.0333 |
| 3 | rs7636754 | 12678725 | G | ADD | 2378 | 0.6309 | 0.2168 | 0.4125 | 0.9648 | -2.125 | 0.03356 |
| 3 | rs6784435 | 12682089 | T | ADD | 2378 | 0.6309 | 0.2168 | 0.4125 | 0.9648 | -2.125 | 0.03356 |
| 3 | rs5746173 | 12686353 | G | ADD | 2378 | 0.6309 | 0.2168 | 0.4125 | 0.9648 | -2.125 | 0.03356 |
| 7 | rs76161861 | 81398644 | A | ADD | 2365 | 0.4885 | 0.3371 | 0.2523 | 0.9459 | -2.125 | 0.03359 |
| 11 | rs117940891 | 1.04E+08 | C | ADD | 2343 | 0.6772 | 0.1837 | 0.4724 | 0.9708 | -2.122 | 0.03388 |
| 11 | rs141568990 | 1.04E+08 | G | ADD | 2343 | 0.6772 | 0.1837 | 0.4724 | 0.9708 | -2.122 | 0.03388 |
| 3 | rs73132351 | 12688284 | G | ADD | 2376 | 0.6315 | 0.2168 | 0.4129 | 0.9659 | -2.12 | 0.034 |
| 3 | rs5746191 | 12658774 | A | ADD | 2377 | 0.6304 | 0.2177 | 0.4114 | 0.966 | -2.119 | 0.03411 |
| 13 | rs8001882 | 28921281 | A | ADD | 2167 | 0.8513 | 0.07609 | 0.7334 | 0.9883 | -2.115 | 0.03442 |
| 7 | rs183801378 | 55154562 | A | ADD | 2342 | 10.71 | 1.121 | 1.19 | 96.42 | 2.115 | 0.03443 |
| 3 | rs6778959 | 12690527 | C | ADD | 2378 | 0.6323 | 0.2168 | 0.4134 | 0.9671 | -2.114 | 0.03448 |
| 15 | rs34895288 | 99496707 | T | ADD | 2302 | 0.8659 | 0.06814 | 0.7577 | 0.9896 | -2.113 | 0.03463 |
| 7 | rs12535578 | 55154586 | G | ADD | 2198 | 0.7809 | 0.1172 | 0.6206 | 0.9826 | -2.11 | 0.03486 |
| 3 | rs6784168 | 12702847 | G | ADD | 2371 | 0.6663 | 0.1926 | 0.4568 | 0.9719 | -2.108 | 0.03504 |
| 7 | rs114465516 | 55150359 | A | ADD | 2365 | 0.6467 | 0.2071 | 0.431 | 0.9703 | -2.105 | 0.03526 |
| 15 | rs2684792 | 99488081 | A | ADD | 2389 | 1.159 | 0.07019 | 1.01 | 1.33 | 2.105 | 0.03528 |
| 13 | rs11149523 | 28995630 | A | ADD | 2314 | 0.8404 | 0.08285 | 0.7144 | 0.9886 | -2.099 | 0.03582 |
| 3 | rs9823121 | 12707277 | T | ADD | 2367 | 0.6642 | 0.195 | 0.4532 | 0.9733 | -2.099 | 0.03584 |
| 4 | rs187262191 | 1.11E+08 | A | ADD | 1930 | 0.3693 | 0.475 | 0.1456 | 0.9369 | -2.097 | 0.03597 |
| 13 | rs9513087 | 28928688 | T | ADD | 2319 | 0.8582 | 0.07297 | 0.7438 | 0.9901 | -2.096 | 0.03606 |
| 11 | rs11226091 | 1.04E+08 | C | ADD | 2327 | 1.159 | 0.07031 | 1.01 | 1.33 | 2.095 | 0.0362 |
| 8 | rs9657467 | 1.42E+08 | C | ADD | 1100 | 0.1523 | 0.8992 | 0.02614 | 0.8875 | -2.093 | 0.03638 |
| 3 | rs9878151 | 12703714 | C | ADD | 2377 | 0.674 | 0.1887 | 0.4656 | 0.9756 | -2.091 | 0.03653 |
| 13 | rs34465640 | 28998799 | G | ADD | 2314 | 0.8424 | 0.08245 | 0.7167 | 0.9902 | -2.08 | 0.03753 |
| 6 | rs139273345 | 1.12E+08 | T | ADD | 2221 | 0.3899 | 0.4539 | 0.1602 | 0.949 | -2.075 | 0.03796 |
| 6 | rs139234015 | 1.12E+08 | A | ADD | 2222 | 0.3905 | 0.4537 | 0.1605 | 0.9501 | -2.073 | 0.0382 |
| 7 | rs61160416 | 55128285 | C | ADD | 2384 | 0.671 | 0.1925 | 0.4602 | 0.9786 | -2.072 | 0.03822 |
| 13 | rs17086708 | 29040660 | G | ADD | 2357 | 1.463 | 0.1835 | 1.021 | 2.096 | 2.072 | 0.03831 |
| 4 | rs34945396 | 55982784 | C | ADD | 1595 | 0.7474 | 0.1406 | 0.5674 | 0.9845 | -2.071 | 0.03833 |
| 13 | rs12429309 | 28879332 | C | ADD | 2388 | 1.163 | 0.07284 | 1.008 | 1.341 | 2.07 | 0.03841 |
| 18 | rs4987716 | 60960979 | A | ADD | 1768 | 0.2946 | 0.5903 | 0.09265 | 0.937 | -2.07 | 0.03843 |
| 7 | rs17335891 | 55131064 | C | ADD | 2325 | 0.8052 | 0.1049 | 0.6556 | 0.989 | -2.065 | 0.03888 |
| 13 | rs11618844 | 29034991 | C | ADD | 2382 | 1.423 | 0.1709 | 1.018 | 1.989 | 2.065 | 0.03893 |
| 5 | rs72818977 | 1.8E+08 | A | ADD | 2286 | 1.323 | 0.1356 | 1.014 | 1.725 | 2.064 | 0.039 |
| 7 | rs73489562 | 1.4E+08 | T | ADD | 2351 | 0.7779 | 0.1217 | 0.6128 | 0.9874 | -2.064 | 0.03903 |
| 17 | rs146113622 | 73351811 | C | ADD | 2336 | 0.08258 | 1.209 | 0.007728 | 0.8825 | -2.063 | 0.03908 |
| 6 | rs78419250 | 41977713 | G | ADD | 2362 | 0.1525 | 0.9124 | 0.0255 | 0.9117 | -2.061 | 0.03927 |
| 7 | rs1558543 | 55135512 | T | ADD | 2365 | 0.658 | 0.2032 | 0.4418 | 0.9799 | -2.06 | 0.03941 |
| 7 | rs28577882 | 1.4E+08 | A | ADD | 2350 | 0.7783 | 0.1217 | 0.6132 | 0.988 | -2.059 | 0.03946 |
| 7 | rs10242534 | 1.4E+08 | C | ADD | 2350 | 0.7783 | 0.1217 | 0.6132 | 0.988 | -2.059 | 0.03946 |
| 5 | rs78795510 | 1.8E+08 | G | ADD | 2101 | 0.1608 | 0.8878 | 0.02822 | 0.9161 | -2.059 | 0.03953 |
| 3 | rs5746214 | 12644753 | G | ADD | 2372 | 0.6341 | 0.2213 | 0.4109 | 0.9785 | -2.058 | 0.03955 |
| 15 | rs12437963 | 99496859 | G | ADD | 2293 | 0.8685 | 0.06853 | 0.7593 | 0.9933 | -2.058 | 0.03958 |
| 4 | rs143507850 | 1.11E+08 | C | ADD | 2300 | 0.3219 | 0.5519 | 0.1091 | 0.9495 | -2.054 | 0.03999 |
| 11 | rs149017693 | 1.04E+08 | C | ADD | 2334 | 0.156 | 0.9053 | 0.02645 | 0.9197 | -2.052 | 0.04013 |
| 8 | rs116935757 | 1.42E+08 | A | ADD | 1667 | 0.3636 | 0.4931 | 0.1383 | 0.9559 | -2.051 | 0.04022 |
| 13 | rs671422 | 29049430 | G | ADD | 2285 | 1.435 | 0.1763 | 1.016 | 2.028 | 2.051 | 0.04031 |
| 3 | rs55977822 | 12704438 | A | ADD | 2367 | 0.6704 | 0.1951 | 0.4574 | 0.9827 | -2.05 | 0.04041 |
| 4 | rs117656018 | 1.11E+08 | T | ADD | 2301 | 0.323 | 0.5517 | 0.1096 | 0.9525 | -2.048 | 0.04054 |
| 7 | rs111599093 | 55125897 | A | ADD | 2376 | 0.6739 | 0.1927 | 0.4619 | 0.9831 | -2.048 | 0.04054 |
| 7 | rs112317270 | 55126160 | C | ADD | 2376 | 0.6739 | 0.1927 | 0.4619 | 0.9831 | -2.048 | 0.04054 |
| 13 | rs9551471 | 29007730 | G | ADD | 2309 | 0.8462 | 0.08155 | 0.7212 | 0.9929 | -2.048 | 0.04058 |
| 17 | rs2217805 | 73334164 | C | ADD | 156 | 1.697 | 0.2584 | 1.023 | 2.817 | 2.047 | 0.04062 |
| 4 | rs12651220 | 1.11E+08 | A | ADD | 2326 | 0.3235 | 0.552 | 0.1097 | 0.9545 | -2.044 | 0.04091 |
| 3 | rs13060685 | 12659392 | G | ADD | 1803 | 0.6984 | 0.1757 | 0.495 | 0.9854 | -2.044 | 0.041 |
| 7 | rs73131843 | 55117116 | G | ADD | 2354 | 0.6929 | 0.1798 | 0.4871 | 0.9858 | -2.04 | 0.04137 |
| 13 | rs10507385 | 28996960 | C | ADD | 2327 | 0.8456 | 0.08228 | 0.7196 | 0.9935 | -2.039 | 0.04146 |
| 13 | rs662560 | 29067336 | C | ADD | 2370 | 1.335 | 0.1419 | 1.011 | 1.763 | 2.035 | 0.04182 |
| 13 | rs646347 | 29044015 | C | ADD | 2291 | 1.422 | 0.1732 | 1.013 | 1.997 | 2.035 | 0.04189 |
| 13 | rs638889 | 29044352 | C | ADD | 2289 | 1.422 | 0.1732 | 1.013 | 1.997 | 2.034 | 0.04199 |
| 13 | rs77374137 | 29030190 | T | ADD | 2360 | 1.461 | 0.1866 | 1.014 | 2.107 | 2.033 | 0.04204 |
| 8 | rs142947254 | 1.42E+08 | G | ADD | 2271 | 0.2653 | 0.6531 | 0.07376 | 0.9543 | -2.032 | 0.0422 |
| 12 | rs12821878 | 1.03E+08 | A | ADD | 2357 | 1.388 | 0.1614 | 1.011 | 1.905 | 2.031 | 0.04228 |
| 5 | rs140492735 | 1.8E+08 | T | ADD | 2286 | 1.318 | 0.136 | 1.009 | 1.72 | 2.029 | 0.04245 |
| 4 | rs11569100 | 1.11E+08 | C | ADD | 2317 | 0.739 | 0.1491 | 0.5518 | 0.9898 | -2.029 | 0.04249 |
| 13 | rs73455407 | 29023682 | G | ADD | 2350 | 1.462 | 0.1872 | 1.013 | 2.11 | 2.029 | 0.04249 |
| 4 | rs79163032 | 55987777 | A | ADD | 2185 | 1.87 | 0.3085 | 1.021 | 3.423 | 2.028 | 0.04251 |
| 13 | rs17086739 | 29051717 | A | ADD | 2331 | 1.408 | 0.1688 | 1.011 | 1.96 | 2.025 | 0.04288 |
| 13 | rs117968512 | 29047362 | A | ADD | 2323 | 1.455 | 0.1853 | 1.012 | 2.092 | 2.024 | 0.04302 |
| 3 | rs6777175 | 12646255 | T | ADD | 2374 | 0.64 | 0.2206 | 0.4153 | 0.9861 | -2.023 | 0.04305 |
| 3 | rs6442319 | 12647029 | A | ADD | 2374 | 0.64 | 0.2206 | 0.4153 | 0.9861 | -2.023 | 0.04305 |
| 3 | rs2290162 | 12647457 | G | ADD | 2374 | 0.64 | 0.2206 | 0.4153 | 0.9861 | -2.023 | 0.04305 |
| 3 | rs5746206 | 12648255 | A | ADD | 2374 | 0.64 | 0.2206 | 0.4153 | 0.9861 | -2.023 | 0.04305 |
| 3 | rs3773349 | 12649732 | C | ADD | 2374 | 0.64 | 0.2206 | 0.4153 | 0.9861 | -2.023 | 0.04305 |
| 15 | rs72754807 | 99506740 | A | ADD | 2290 | 1.875 | 0.3109 | 1.019 | 3.449 | 2.022 | 0.04318 |
| 7 | rs75726430 | 55148154 | G | ADD | 2360 | 0.6542 | 0.2101 | 0.4334 | 0.9876 | -2.019 | 0.04344 |
| 13 | rs9554330 | 29003444 | A | ADD | 2390 | 0.8501 | 0.08053 | 0.726 | 0.9955 | -2.016 | 0.04377 |
| 7 | rs17172436 | 55148715 | C | ADD | 2363 | 0.6598 | 0.2065 | 0.4401 | 0.989 | -2.014 | 0.04405 |
| 15 | rs72752895 | 99497467 | T | ADD | 2262 | 0.8694 | 0.06949 | 0.7587 | 0.9963 | -2.014 | 0.04406 |
| 11 | rs11226075 | 1.04E+08 | C | ADD | 2169 | 0.8655 | 0.07177 | 0.7519 | 0.9962 | -2.013 | 0.04411 |
| 13 | rs9513070 | 28879839 | G | ADD | 2378 | 1.162 | 0.07452 | 1.004 | 1.344 | 2.012 | 0.04426 |
| 7 | rs57132241 | 1.16E+08 | A | ADD | 2329 | 1.392 | 0.1649 | 1.008 | 1.924 | 2.007 | 0.04477 |
| 17 | rs55717377 | 37850569 | T | ADD | 1377 | 0.8181 | 0.1001 | 0.6724 | 0.9954 | -2.006 | 0.0449 |
| 3 | rs13070228 | 12706914 | A | ADD | 2377 | 0.685 | 0.1887 | 0.4732 | 0.9915 | -2.005 | 0.04497 |
| 13 | rs659413 | 29043435 | C | ADD | 2349 | 1.424 | 0.1764 | 1.008 | 2.011 | 2.003 | 0.04522 |
| 15 | rs77605019 | 99387367 | G | ADD | 2257 | 1.634 | 0.2458 | 1.01 | 2.646 | 1.999 | 0.04564 |
| 4 | rs151262481 | 1.58E+08 | C | ADD | 2173 | 4.822 | 0.7875 | 1.03 | 22.57 | 1.998 | 0.04577 |
| 4 | rs9991904 | 1.11E+08 | G | ADD | 2323 | 0.7431 | 0.1489 | 0.5549 | 0.995 | -1.994 | 0.04616 |
| 15 | rs74785122 | 99378882 | A | ADD | 2259 | 1.632 | 0.2458 | 1.008 | 2.643 | 1.994 | 0.0462 |
| 7 | rs17289533 | 55204109 | A | ADD | 2333 | 0.2141 | 0.7732 | 0.04704 | 0.9747 | -1.993 | 0.04625 |
| 5 | rs3797102 | 1.8E+08 | G | ADD | 2390 | 1.149 | 0.06975 | 1.002 | 1.317 | 1.991 | 0.04646 |
| 13 | rs78602116 | 29028661 | A | ADD | 2363 | 1.445 | 0.1849 | 1.006 | 2.076 | 1.991 | 0.0465 |
| 13 | rs76839733 | 29028682 | G | ADD | 2363 | 1.445 | 0.1849 | 1.006 | 2.076 | 1.991 | 0.0465 |
| 7 | rs3735062 | 55153237 | A | ADD | 2240 | 0.6426 | 0.2223 | 0.4156 | 0.9934 | -1.99 | 0.04664 |
| 5 | rs7709359 | 1.8E+08 | G | ADD | 1845 | 1.163 | 0.07613 | 1.002 | 1.35 | 1.986 | 0.04699 |
| 3 | rs5746187 | 12659419 | G | ADD | 2377 | 0.6494 | 0.2174 | 0.4241 | 0.9944 | -1.986 | 0.04704 |
| 7 | rs10488140 | 55138388 | T | ADD | 2319 | 0.7337 | 0.1565 | 0.5399 | 0.997 | -1.979 | 0.04782 |
| 7 | rs151015237 | 81389727 | C | ADD | 2314 | 0.06838 | 1.356 | 0.004794 | 0.9754 | -1.978 | 0.0479 |
| 15 | rs74032119 | 99488362 | A | ADD | 2313 | 0.5219 | 0.3291 | 0.2739 | 0.9947 | -1.976 | 0.04816 |
| 15 | rs114962406 | 99488579 | A | ADD | 2313 | 0.5219 | 0.3291 | 0.2739 | 0.9947 | -1.976 | 0.04816 |
| 13 | rs11618340 | 29066152 | C | ADD | 2368 | 1.324 | 0.1422 | 1.002 | 1.749 | 1.973 | 0.04845 |
| 18 | rs7242542 | 60901926 | T | ADD | 2243 | 1.206 | 0.095 | 1.001 | 1.453 | 1.973 | 0.04853 |
| 15 | rs939626 | 99493176 | C | ADD | 2159 | 1.171 | 0.08023 | 1.001 | 1.37 | 1.968 | 0.04906 |
| 2 | rs145951686 | 39223403 | T | ADD | 2102 | 0.3992 | 0.4668 | 0.1599 | 0.9967 | -1.967 | 0.04919 |
| 7 | rs6464036 | 1.4E+08 | T | ADD | 2365 | 0.79 | 0.1199 | 0.6246 | 0.9993 | -1.966 | 0.04934 |
| 7 | rs917880 | 55162011 | T | ADD | 2385 | 0.8416 | 0.08784 | 0.7085 | 0.9998 | -1.963 | 0.04969 |
| 4 | rs6840890 | 1.11E+08 | C | ADD | 2330 | 0.4094 | 0.4552 | 0.1678 | 0.999 | -1.962 | 0.04974 |
| 4 | rs140001952 | 1.11E+08 | C | ADD | 2330 | 0.4095 | 0.4552 | 0.1678 | 0.9993 | -1.962 | 0.04981 |
