# Supplementary material for: RTK/ERK Pathway under Natural Selection Associated with Prostate Cancer
Source: PLoS One. 2013 Nov 4;8(11):e78254. doi: 10.1371/journal.pone.0078254 (PMC3817240; doi:10.1371/journal.pone.0078254)
Supplement: Table S2 — Expression of quantitative trait loci (eQTLs) analysis with Geneva in three different databases (HapMapIII, MuTHER healthy female twins and Geneva GenCord individuals). *the results presented in the tables were significant collecting from the total eQTLs analysis for all SNPs in table 3. (DOC) [file pone.0078254.s002.doc]

Table S2 expression of quantitative trait loci (eQTLs) analysis with Geneva in three different databases (HapMapIII, MuTHER healthy female twins and Geneva GenCord individuals).

*the results presented in the tables were significant collecting from the total eQTLs analysis for all SNPs in table 3

| SNP | Gene | Probe ID | Probe start | HapMap3 (*P*) | | | | cell types(*P*) | | | twin1 | | | twin2 | | |
| --- | --- | --- | --- | --- | --- | --- | --- | --- | --- | --- | --- | --- | --- | --- | --- | --- |
|  |  |  |  | CEU | CHB | JPT | YRI | fibroblast | LCL | T-cell | adipose | LCL | skin | adipose | LCL | skin |
| rs11238349 (EGFR) | VSTM2A | ILMN_1765620 | 54580485 | 0.7265 | 0.438 | 0.9339 |  | 0.5584 | 0.3863 | 0.4411 | 0.1012 | **0.0108** | 0.2954 | **0.0197** | 0.5872 | 0.9023 |
|  | EGFR | ILMN_1728858 | 55055585 | **0.013** | 0.7056 | 0.617 |  | 0.2234 | 0.6328 | 0.618 | 0.6926 | 0.9136 | 0.1114 | 0.1087 | 0.7405 | 0.4769 |
|  | LANCL2 | ILMN_1708009 | 55404701 | **0.0091** | **0.0094** | 0.7795 |  | 0.5 | 0.06 | 0.9493 | 0.8159 | 0.6077 | 0.1331 | 0.7027 | 0.7696 | 0.8254 |
|  | GBAS | ILMN_1778611 | 56001470 | 0.3856 | 0.1632 | 0.2999 |  | 0.4635 | 0.8561 | 0.9279 | 0.7392 | 0.3937 | 0.5329 | 0.8919 | 0.2701 | **0.0419** |
|  | CCT6A | ILMN_1722502 | 56087322 | 0.5039 | 0.0588 | 0.3162 |  | 0.8094 | 0.1778 | 0.2251 | 0.6202 | 0.2583 | 0.7261 | 0.4507 | 0.244 | **0.019** |
|  | SUMF2 | ILMN_1685371 | 56100793 | 0.9797 | 0.8062 | 0.1224 |  | **0.015** | 0.8084 | 0.4195 | 0.1682 | 0.7672 | **0.0128** | 0.6526 | 0.2724 | 0.3543 |
|  | EGFR | ILMN_1755535 | 55056358 |  |  |  |  | 0.2015 | **0.0014** | 0.9953 | 0.2804 | 0.536 | 0.7656 | 0.458 | 0.7542 | 0.9865 |
|  | ZNF713 | ILMN_1697289 | 55923736 |  |  |  |  | 0.5778 | **0.0017** | 0.8172 | 0.6937 | 0.5668 | 0.4488 | 0.7223 | 0.7594 | 0.6167 |
|  | FKBP9L | ILMN_1709650 | 55726571 |  |  |  |  |  |  |  | 0.4193 | 0.9506 | **0.0202** | 0.1785 | 0.5728 | 0.1383 |
| rs17172438 (EGFR) | EGFR | ILMN_1728858 | 55055585 | 0.4575 | 0.7852 | 0.5994 | **0.0382** |  |  |  |  |  |  |  |  |  |
|  | CCT6A | ILMN_1681160 | 56087322 | 0.5729 | 0.9944 | 0.5732 | **0.0291** |  |  |  |  |  |  |  |  |  |
| rs984654 (EGFR) | EGFR | ILMN_1755535 | 55056358 |  |  |  |  | 0.0609 | **10-4** | 0.8675 | 0.2762 | 0.3147 | 0.5368 | 0.1502 | 0.1106 | 0.1149 |
|  | LANCL2 | ILMN_1708009 | 55404701 | **0.0496** | **0.0093** | 0.7108 |  | 0.3294 | 0.1131 | 0.4136 | 0.4861 | 0.0835 | 0.5568 | **0.049** | 0.8933 | 0.3305 |
|  | ZNF713 | ILMN_1697289 | 55923736 |  |  |  |  | 0.7757 | **0.017** | 0.8543 | 0.8879 | 0.8907 | 0.395 | 0.2407 | 0.3244 | 0.9538 |
|  | SUMF2 | ILMN_1685371 | 56100686 | 0.6847 | 0.6128 | 0.4846 |  | 0.0336 | 0.2951 | 0.9408 | 0.1313 | 0.6731 | 0.1329 | 0.9395 | 0.8401 | 0.5722 |
|  | SEPT14 | ILMN_1798053 | 55895167 | 0.9561 | 0.0849 | 0.2517 |  | 0.3393 | 0.3967 | 0.5716 | 0.9608 | 0.5644 | 0.9408 | **0.0176** | 0.1868 | 0.7869 |
|  | SEPT14 | ILMN_2055925 | 55895336 |  |  |  |  | 0.1112 | 0.7706 | 0.7751 | 0.6216 | 0.4454 | 0.699 | 0.9549 | 0.7771 | 0.0331 |
|  | SEC61G | ILMN_1787026 | 54794387 | 0.7652 | 0.6319 | 0.8535 |  | 0.2666 | **0.0437** | 0.8577 | 0.8363 | 0.7052 | 0.9591 | 0.5822 | 0.3349 | 0.1641 |
|  | SEC61G | ILMN_2367020 | 54794329 |  |  |  |  | 0.195 | **0.045** | 0.4639 | 0.8928 | 0.2621 | 0.8706 | 0.6003 | 0.3574 | 0.1571 |
|  |  | ILMN_2226955 | 55606185 |  |  |  |  |  |  |  | **0.0248** | 0.0835 | 0.4596 | **0.0159** | 0.6524 | 0.1153 |
| rs6978771 (EGFR) | LANCL2 | ILMN_1708009 | 55404701 | **0.0553** | **0.0028** | 0.9485 |  |  |  |  |  |  |  |  |  |  |
|  | CCT6A | ILMN_1722502 | 56087322 | 0.4151 | **0.0285** | 0.7207 |  |  |  |  |  |  |  |  |  |  |
| rs11773818 (EGFR) | LANCL2 | ILMN_1708009 | 55404701 | **0.0507** | **0.0242** | 0.4191 |  | 0.2182 | 0.4283 | 0.3787 | 0.5954 | 0.1034 | 0.713 | **0.0383** | 0.7895 | 0.224 |
|  | SEPT14 | ILMN_1798053 | 55880589 | 0.8277 | **0.0312** | 0.9047 |  | 0.4167 | 0.3546 | 0.4876 | 0.9532 | 0.1864 | 0.8877 | **0.0285** | 0.3448 | 0.6583 |
|  | EGFR | ILMN_1755535 | 55056358 |  |  |  |  | **0.0675** | **<10-4** | 0.6259 | 0.2513 | 0.3838 | 0.4107 | 0.2582 | 0.3122 | 0.1065 |
|  | ZNF713 | ILMN_1697289 | 55923736 |  |  |  |  | 0.9034 | **0.0149** | 0.7857 | 0.8313 | 0.6817 | 0.4014 | 0.3507 | 0.5212 | 0.8555 |
|  | SEPT14 | ILMN_2055925 | 55895336 |  |  |  |  | 0.1279 | 0.6225 | 0.6597 | 0.482 | 0.4107 | 0.8996 | 0.7387 | 0.7973 | **0.031** |
|  |  | ILMN_2226955 | 55606185 |  |  |  |  |  |  |  | **0.0281** | **0.0535** | 0.3058 | **0.0304** | 0.8931 | 0.1817 |
| rs17172432 (EGFR) | EGFR | ILMN_1798975 | 55055585 | 0.151 | 0.2501 | **0.0424** | 0.6499 |  |  |  |  |  |  |  |  |  |
|  | LANCL2 | ILMN_1708009 | 55404701 | 0.6894 | **0.0336** | 0.8748 | 0.351 |  |  |  |  |  |  |  |  |  |
| rs6494584 | SMAD3 | ILMN_1682738 | 65151186 |  | 0.8115 | 0.7475 | **0.0104** |  |  |  |  |  |  |  |  |  |
|  |  | ILMN_1655311 | 65600866 |  | 0.732 | 0.5192 | **0.0288** |  |  |  |  |  |  |  |  |  |
|  | MAP2K5 | ILMN_1718129 | 65720191 |  | 0.8349 | 0.838 | **0.0249** |  |  |  |  |  |  |  |  |  |
| rs7115260 | IGHMBP2 | ILMN_1801909 | 68431533 | 0.5822 |  | 0.2261 | **0.0254** |  |  |  | 0.2146 | 0.8907 | 0.1403 | 0.1385 | 0.9123 | 0.985 |
|  | TPCN2 | ILMN_1726873 | 68575690 | 0.3311 |  | 0.2663 | 0.0751 |  |  |  | 0.0963 | 0.396 | 0.7264 | **0.0348** | 0.1437 | 0.683 |
|  | FGF3 | ILMN_1723227 | 69341843 | 0.5779 |  | 0.3285 | 0.4737 |  |  |  | 0.0428 | 0.3236 | 0.5136 | 0.7869 | 0.2979 | 0.3093 |
|  | FGF4 | ILMN_1677456 | 69298482 | 0.4218 |  | 0.1286 | **0.0256** |  |  |  | 0.3884 | 0.8952 | 0.7751 | 0.7454 | 0.8971 | 0.5005 |
|  |  | ILMN_1703579 | 69180884 | 0.3564 |  | 0.4221 | **0.0011** |  |  |  | **0.0432** | 0.4505 | **0.0454** | 0.4971 | 0.5079 | 0.7103 |
|  | MRGPRF | ILMN_1657502 | 68535436 | **0.0355** |  | 0.8892 | **0.0133** |  |  |  |  |  |  |  |  |  |
|  | CPT1A | ILMN_1687589 | 68363316 | **0.0486** |  | 0.5328 | **0.0263** |  |  |  | 0.9098 | 0.948 | 0.0858 | 0.6874 | 0.2359 | 0.9651 |
|  | PPFIA1 | ILMN_2382127 | 69797952 |  |  |  |  |  |  |  | 0.0849 | 0.381 | 0.0153 | 0.1567 | 0.7637 | 0.0918 |
| rs2255648 (MKRN2) | SYN2 | ILMN_1781060 | 12023108 | 0.6152 | **0.0356** | 0.0576 | **0.0434** |  |  |  | **0.0127** | 0.4518 | 0.1567 | 0.4701 | 0.9875 | 0.9852 |
|  | MKRN2 | ILMN_1663727 | 12576134 | **0.0022** | 0.6175 | **7*10-4** | **4*10-4** |  |  |  |  |  |  |  |  |  |
|  | CAND2 | ILMN_1746865 | 12813382 | 0.8459 | 0.9854 | 0.9833 | **0.0323** |  |  |  |  |  |  |  |  |  |
|  | HDAC11 | ILMN_1684690 | 13498483 | 0.5802 | 0.8066 | 0.3974 | **0.0124** |  |  |  | 0.4221 | 0.407 | 0.2459 | 0.1153 | 0.2352 | 0.171 |
|  | FBLN2 | ILMN_1774602 | 13567940 | 0.7703 | **0.0012** | 0.834 | 0.926 |  |  |  | 0.3102 | 0.6778 | 0.518 | 0.4807 | 0.9867 | 0.9437 |
|  | NUP210 | ILMN_1784467 | 13429968 | 0.2413 | 0.3039 | 0.0538 | **0.0318** |  |  |  | 0.1381 | 0.2123 | 0.2591 | 0.9614 | 0.6673 | 0.0611 |
|  | RPL32 | ILMN_1663799 | 12857499 | 0.8797 | 0.3786 | 0.0095 | 0.2289 |  |  |  | 0.7844 | **0.0201** | 0.7478 | 0.5275 | 0.1446 | 0.3067 |
|  | RPL32 | ILMN_1798636 | 12857499 | 0.7643 | 0.8799 | 0.5016 | **0.0051** |  |  |  | 0.7138 | 0.5074 | 0.103 | 0.3043 | 0.9547 | 0.4233 |
|  | RAF1 | ILMN_1813489 | 12677695 | **0.0378** | 0.8164 | **0.0101** | **0.0033** |  |  |  | 0.4662 | 0.4105 | 0.2719 | 0.0751 | 0.9047 | 0.9986 |
|  | MKRN2 | ILMN_2056760 | 12575817 |  |  |  |  |  |  |  | **0.012** | 0.0672 | 0.8338 | 0.0561 | 0.9694 | 0.7179 |
| rs1542848 (MKRN2) | TSEN2 | ILMN_1746393 | 12503182 | 0.2781 | 0.6163 | 0.6097 | 0.439 |  |  |  | 0.1003 | 0.5069 | 0.7401 | **0.0298** | 0.518 | 0.8449 |
|  | MKRN2 | ILMN_1663727 | 12576134 | **0.0020** | 0.7293 | **10-4** | 0.6012 |  |  |  |  |  |  |  |  |  |
|  | RPL32 | ILMN_1663799 | 12857499 | 0.9395 | 0.5454 | **0.0056** | 0.5762 |  |  |  |  |  |  |  |  |  |
|  | RAF1 | ILMN_1813489 | 12677695 | **0.0163** | 0.974 | **0.0065** | 0.5592 |  |  |  |  |  |  |  |  |  |
| rs2633442 (MKRN2) | SYN2 | ILMN_1781060 | 12023108 | 0.5945 | **0.0367** | 0.0946 | 0.2069 |  |  |  |  |  |  |  |  |  |
|  | MKRN2 | ILMN_1663727 | 12576134 | **0.0034** | 0.6092 | **<10-4** | 0.314 |  |  |  |  |  |  |  |  |  |
|  | RAF1 | ILMN_1813489 | 12677695 | **0.044** | 0.8166 | 0.0065 | 0.7773 |  |  |  |  |  |  |  |  |  |
| rs2442802 (MKRN2) | SYN2 | ILMN_1781060 | 12023108 | 0.5995 | **0.0318** | 0.0904 | 0.1575 |  |  |  | **0.0087** | 0.3264 | 0.3549 | 0.8064 | 0.9144 | 0.9737 |
|  | PPARG | ILMN_1800225 | 12305446 | 0.6408 | 0.1433 | 0.3859 | 0.7123 | 0.2912 | 0.9174 | **0.012** | 0.9996 | 0.6621 | 0.7766 | 0.3832 | 0.5188 | 0.6253 |
|  | PPARG | ILMN_1687612 | 12305446 | 0.9677 | 0.1433 | 0.1974 | 0.7209 | 0.4367 | 0.6514 | **0.0041** | 0.3491 | 0.1299 | 0.482 | **0.0451** | 0.1117 | 0.3475 |
|  | MKRN2 | ILMN_1663727 | 12576134 | **0.0035** | 0.658 | **10-4** | 0.2066 |  |  |  |  |  |  |  |  |  |
|  | FBLN2 | ILMN_1774602 | 13567940 | 0.759 | **0.0033** | 0.7541 | 0.8526 |  |  |  |  |  |  |  |  |  |
|  | RPL32 | ILMN_1663799 | 12857499 | 0.677 | 0.3395 | 0.0061 | 0.5057 | 0.1368 | 0.62 | 0.8076 | 0.8798 | **0.0175** | 0.5534 | 0.5326 | 0.2209 | 0.3413 |
|  | RAF1 | ILMN_1813489 | 12677695 | **0.0413** | 0.8463 | 0.0078 | 0.6313 | 0.5649 | 0.4849 | 0.9382 | 0.322 | 0.4946 | 0.2452 | 0.0796 | 0.8389 | 0.5984 |
|  | MKRN2 | ILMN_2056760 | 12575817 |  |  |  |  | 0.0822 | 0.3147 | **0.0168** | **0.0214** | 0.0385 | 0.9492 | 0.0592 | 0.9364 | 0.937 |
| rs10488140 (EGFR) | MRPS17 | ILMN_1804851 | 55987450 | 0.3362 | 0.2637 |  | 0.8687 | 0.2003 | 0.5642 | 0.5942 | 0.9897 | 0.3011 | 0.1765 | 0.5319 | 0.8078 | **0.0285** |
|  | GBAS | ILMN_1778611 | 56001470 | 0.784 | 0.0787 |  | 0.6118 | 0.5792 | 0.4477 | 0.2146 | 0.3812 | 0.8 | 0.3177 | 0.2163 | **0.038** | **0.0248** |
|  | PSPH | ILMN_1776105 | 56085498 | 0.4636 | 0.1406 |  | 0.9317 | 0.5455 | 0.7963 | 0.3195 | 0.3485 | 0.6505 | 0.656 | 0.0041 | 0.3803 | 0.3463 |
|  | SEPT14 | ILMN_1798053 | 55880589 | 0.0936 | 0.1392 |  | 0.7347 | 0.5582 | 0.3741 | 0.7573 | 0.5761 | 0.4086 | **0.0068** | 0.8441 | 0.7349 | 0.6372 |
|  | FKBP9L | ILMN_2089977 | 55716483 |  |  |  |  |  |  |  | 0.447 | 0.6296 | 0.7772 | 0.161 | **0.0355** | 0.1008 |
| rs2005219 | QPRT | ILMN_1669211 | 29599143 | 0.4899 |  | **0.0276** |  |  |  |  |  |  |  |  |  |  |
|  |  | ILMN_1670376 | 29698440 | 0.7423 |  | 0.2388 |  |  |  |  | 0.0704 | 0.159 | **0.0155** | 0.7956 | 0.999 | 0.6042 |
|  | PPP4C | ILMN_1750364 | 29995998 | **0.0479** |  | **9*10-4** |  |  |  |  |  |  |  |  |  |  |
|  | ZNF771 | ILMN_1794753 | 30331779 | 0.4676 |  | **0.0087** |  |  |  |  | 0.7776 | 0.5309 | **0.0383** | 0.5349 | 0.7053 | 0.289 |
|  | FBRS | ILMN_1752559 | 30582082 | 0.7702 |  | **0.0397** |  |  |  |  |  |  |  |  |  |  |
|  |  | ILMN_1750641 | 30627727 | 0.9818 |  | 0.299 |  |  |  |  | 0.5606 | 0.2864 | **0.0309** | **0.05** | 0.0192 | 0.425 |
|  | RNF40 | ILMN_1808712 | 30685013 | 0.6438 |  | 0.3526 |  |  |  |  | 0.3664 | 0.6141 | 0.9149 | 0.9456 | **0.0223** | 0.2706 |
|  | CTF1 | ILMN_1661804 | 30813247 | 0.7619 |  | 0.9621 |  |  |  |  | 0.5051 | 0.5745 | 0.7762 | 0.0106 | 0.6249 | 0.5024 |
|  | ORAI3 | ILMN_1736628 | 30869858 | 0.2981 |  | 0.0518 |  |  |  |  | **0.0498** | **0.0423** | 0.0874 | 0.6914 | 0.3064 | 0.5159 |
|  | HSD3B7 | ILMN_1653042 | 30905792 | 0.0526 |  | 0.4358 |  |  |  |  | 0.7518 | **0.0209** | 0.4621 | 0.3637 | **0.0388** | 0.514 |
|  | ZNF764 | ILMN_1727938 | 30474697 | 0.7838 |  | **0.0271** |  |  |  |  | 0.8202 | 0.9208 | 0.5569 | 0.9812 | 0.0803 | 0.3594 |
|  | ZNF747 | ILMN_1762098 | 30451555 | 0.8053 |  | 0.5657 |  |  |  |  | 0.7239 | 0.4047 | **0.0155** | 0.927 | 0.8557 | 0.9125 |
|  | ZNF768 | ILMN_1791820 | 30443476 | 0.7797 |  | 0.0535 |  |  |  |  | **0.0070** | 0.6286 | 0.8636 | 0.8987 | 0.0982 | 0.2101 |
|  | MAPK3 | ILMN_1812747 | 30040394 | 0.0666 |  | **<10-4** |  |  |  |  |  |  |  |  |  |  |
|  | GDPD3 | ILMN_1774901 | 30031512 | **0.0129** |  | 0.4623 |  |  |  |  | 0.4658 | 0.9145 | 0.0032 | 0.2494 | **7*10-4** | 0.4993 |
|  | ZNF785 | ILMN_1676459 | 30502034 | 0.5574 |  | 0.6882 |  |  |  |  | 0.6436 | 0.4633 | 0.8053 | 0.4108 | **0.0026** | 0.5228 |
|  | TBX6 | ILMN_1692095 | 30009459 | 0.8122 |  | **0.0399** |  |  |  |  | 0.4278 | 0.0523 | 0.1954 | 0.6351 | 0.5116 | 0.4859 |
|  | SEZ6L2 | ILMN_1704746 | 29816847 | 0.5084 |  | **0.0283** |  |  |  |  |  |  |  |  |  |  |
|  | GIYD1 | ILMN_1664993 | 29373807 |  |  |  |  |  |  |  | 0.7645 | 0.686 | 0.2091 | 0.2155 | 0.8433 | **0.0132** |
|  | SPN | ILMN_1660315 | 29581806 |  |  |  |  |  |  |  | 0.5124 | 0.1823 | 0.3261 | 0.58 | **0.0174** | 0.3198 |
|  | SPN | ILMN_1801040 | 29588269 |  |  |  |  |  |  |  | 0.1522 | **0.0125** | 0.4385 | 0.0782 | 0.0971 | 0.6484 |
|  | TAOK2 | ILMN_2298588 | 29897194 |  |  |  |  |  |  |  | 0.4492 | **0.0254** | 0.6106 | **0.0015** | 0.2381 | 0.2436 |
|  | ALDOA | ILMN_2251253 | 29971983 |  |  |  |  |  |  |  | **0.014** | 0.7147 | 0.1235 | 0.2375 | 0.3854 | 0.322 |
|  | ALDOA | ILMN_1736700 | 29972048 |  |  |  |  |  |  |  | 0.9002 | 0.8475 | 0.4655 | **0.0229** | **0.0182** | 0.6442 |
|  | GIYD2 | ILMN_2368575 | 30113639 |  |  |  |  |  |  |  | 0.3634 | 0.6198 | **0.0486** | 0.2627 | 0.9489 | 0.379 |
|  | SULT1A3 | ILMN_1815283 | 30113932 |  |  |  |  |  |  |  | 0.5353 | 0.3886 | 0.2366 | **0.0447** | 0.1511 | 0.7248 |
|  | VKORC1 | ILMN_1739946 | 31013929 |  |  |  |  |  |  |  | 0.7712 | 0.8266 | 0.9705 | **0.0023** | 0.7505 | 0.5874 |
|  |  | ILMN_2133187 | 31005787 |  |  |  |  |  |  |  | 0.0957 | 0.4221 | **0.013** | 0.7931 | 0.8155 | 0.6559 |
|  |  | ILMN_2179726 | 30679302 |  |  |  |  |  |  |  | 0.1041 | 0.2596 | **0.0125** | **0.0497** | 0.2715 | 0.4095 |
|  | SEPT1 | ILMN_2415162 | 30300454 |  |  |  |  |  |  |  | 0.1199 | 0.6073 | **0.0477** | 0.6554 | **0.0246** | 0.2735 |
|  | MAPK3 | ILMN_1667260 | 30040351 |  |  |  |  |  |  |  | 0.4326 | 0.1839 | 0.2208 | 0.6247 | **0.026** | 0.5074 |
|  | MAPK3 | ILMN_2402341 | 30040358 |  |  |  |  |  |  |  | 0.4992 | 0.1552 | 0.8498 | 0.1183 | **0.0102** | 0.6576 |
|  | FAM57B | ILMN_1655498 | 29943312 |  |  |  |  |  |  |  | 0.2154 | 0.1913 | 0.5554 | **0.0027** | 0.8976 | 0.8134 |
|  | DOC2A | ILMN_2172507 | 29928636 |  |  |  |  |  |  |  | 0.6285 | 0.4785 | 0.4701 | **0.0403** | 0.8732 | 0.3103 |
|  | DOC2A | ILMN_1812477 | 29929072 |  |  |  |  |  |  |  | 0.7498 | 0.5991 | 0.6958 | **<10-4** | 0.1307 | 0.4167 |
|  | SEZ6L2 | ILMN_1800873 | 29804492 |  |  |  |  |  |  |  | **0.012** | 0.8776 | 0.4957 | **0.0156** | 0.9981 | 0.5825 |
|  | C16orf54 | ILMN_1681032 | 29663460 |  |  |  |  |  |  |  | 0.1637 | 0.4789 | 0.2109 | 0.9921 | **0.032** | 0.1839 |
|  | BOLA2 | ILMN_1656382 | 29373435 |  |  |  |  |  |  |  | 0.5692 | 0.968 | 0.3266 | **0.0043** | 0.4699 | 0.1543 |
|  | BOLA2 | ILMN_2298511 | 29372822 |  |  |  |  |  |  |  | **0.0476** | 0.2593 | 0.6652 | 0.5164 | 0.9693 | 0.5964 |
